# *Ixodes ricinus* and Its Endosymbiont *Midichloria mitochondrii*: A Comparative Proteomic Analysis of Salivary Glands and Ovaries

**DOI:** 10.1371/journal.pone.0138842

**Published:** 2015-09-23

**Authors:** Monica Di Venere, Marco Fumagalli, Alessandra Cafiso, Leone De Marco, Sara Epis, Olivier Plantard, Anna Bardoni, Roberta Salvini, Simona Viglio, Chiara Bazzocchi, Paolo Iadarola, Davide Sassera

**Affiliations:** 1 Department of Molecular Medicine, University of Pavia, Pavia, Italy; 2 Department of Biology and Biotechnology, University of Pavia, Pavia, Italy; 3 Department of Veterinary Science and Public Health, University of Milan, Milan, Italy; 4 School of Bioscience and Veterinary Medicine, University of Camerino, Camerino, Italy; 5 INRA, UMR1300 Biologie, Epidémiologie et Analyse de Risque en santé animale, CS 40706, Nantes, France; 6 LUNAM Université, Oniris, Ecole nationale vétérinaire, agroalimentaire et de l’alimentation Nantes-Atlantique, UMR BioEpAR, Nantes, France; University of Minnesota, UNITED STATES

## Abstract

Hard ticks are hematophagous arthropods that act as vectors of numerous pathogenic microorganisms of high relevance in human and veterinary medicine. *Ixodes ricinus* is one of the most important tick species in Europe, due to its role of vector of pathogenic bacteria such as *Borrelia burgdorferi* and *Anaplasma phagocytophilum*, of viruses such as tick borne encephalitis virus and of protozoans as *Babesia* spp. In addition to these pathogens, *I*. *ricinus* harbors a symbiotic bacterium, *Midichloria mitochondrii*. This is the dominant bacteria associated to *I*. *ricinus*, but its biological role is not yet understood. Most *M*. *mitochondrii* symbionts are localized in the tick ovaries, and they are transmitted to the progeny. *M*. *mitochondrii* bacteria have however also been detected in the salivary glands and saliva of *I*. *ricinus*, as well as in the blood of vertebrate hosts of the tick, prompting the hypothesis of an infectious role of this bacterium. To investigate, from a proteomic point of view, the tick *I*. *ricinus* and its symbiont, we generated the protein profile of the ovary tissue (OT) and of salivary glands (SG) of adult females of this tick species. To compare the OT and SG profiles, 2-DE profiling followed by LC-MS/MS protein identification were performed. We detected 21 spots showing significant differences in the relative abundance between the OT and SG, ten of which showed 4- to 18-fold increase/decrease in density. This work allowed to establish a method to characterize the proteome of *I*. *ricinus*, and to detect multiple proteins that exhibit a differential expression profile in OT and SG. Additionally, we were able to use an immunoproteomic approach to detect a protein from the symbiont. Finally, the method here developed will pave the way for future studies on the proteomics of *I*. *ricinus*, with the goals of better understanding the biology of this vector and of its symbiont *M*. *mitochondrii*.

## Introduction

Vector-borne diseases are among the leading causes of sanitary concern worldwide, being responsible for millions of deaths every year [1]. Although most vectors (such as mosquitoes) mainly exert their toll on developing countries, ticks are also widespread in the Northern hemisphere and are indeed considered the most important disease vectors in Europe and North America [2]. Many species of this order of obligate hematophagous parasites, the Ixodida, are capable of transmitting numerous viral, bacterial, and protozoan pathogens through the blood meal. Hard ticks in particular are dangerous vectors and, among them, the sheep tick *Ixodes ricinus* is one of the most relevant species. The importance of *I*. *ricinus* is due to its wide area of distribution (i.e. Europe and Northern Africa), to its low host specificity and capacity to parasitize humans, and to its central role in the transmission of multiple infectious agents [3]. *Borrelia* spp, the causative agent of Lyme disease, is possibly the most important microorganism vectored by *I*. *ricinus*. This bacterium is responsible for hundreds of thousands of novel infections each year and its role in multiple chronic pathologies is currently being investigated [4]. Additionally, *I*. *ricinus* is capable of transmitting numerous other bacteria, such as *Rickettsia* spp. and *Ehrlichia* spp., but also the flavivirus responsible for Tick Borne Encephalitis and the etiological protozoan agents of babesiosis [5].

In addition to the aforementioned pathogens, *I*. *ricinus* harbors a recently described bacterium named *Midichloria mitochondrii* [6]. The importance of this bacterial presence is testified by its prevalence in the field, as 100% of females, eggs and immatures of *I*. *ricinus* harbor *M*. *mitochondrii* bacteria [7]. Additionally, the bacterial load has been found to be important, with up to 15 million bacteria counted in one single tick, localized mainly in the ovaries or ovarian primordia [8, 9]. As all member of the bacterial order Rickettsiales, *M*. *mitochondrii* is intracellular, however its uniqueness lays in that it was observed inside the mitochondria of the host ovarian cells [9]. Finally, *M*. *mitochondrii* has also been detected in the salivary glands of adult females of *I*. *ricinus*, as well as in the blood of mammalian tick hosts [10, 11]. However, investigations on its potential role as infectious agent have never provided adequate answers concerning the possible link to pathological effects. As a consequence, new tools are required for answering this question.

With the advent of proteomics, the screening of proteins as potential biomarkers has achieved important progresses. Detection and identification of proteins in different organs/tissues, with the aim of understanding whether they represent an attractive tool for monitoring alterations in these districts, is currently an area of increasing interest. Recently, studies have been focused on the characterization of *I*. *ricinus* salivary glands and midgut proteomes, in a much-needed effort to better understand the role of these organs, fundamental in the tick bite and metabolism [12, 13].

Our plan was then to expand the knowledge of *I*. *ricinus* protein profiles by applying two-dimensional electrophoresis (2-DE) as a tool for comparing the protein pattern of the ovary with that of salivary glands (i.e. the sialome). The first goal of this study is to give insight into the process of oogenesis, central to the tick life cycle. Additionally we planned to provide clues on the symbiotic relationship between *I*. *ricinus* and its symbiont *M*. *mitochondrii*, which is highly prevalent in the ovaries. Moreover, to seek the best possible protocol for future studies on *I*. *ricinus* proteomics, we focused on a careful optimization of the proteomic analysis pipeline.

## Materials and Methods

### Ethics Statement

*I*. *ricinus* ticks were collected from roe-deer (*Capreolus capreolus*) in the Chize forest (Northern France) in February 2014, in strict accordance with the recommendations in the french National charter on the ethics of animal experimentation and the DIRECTIVE 2010/63/EU OF THE EUROPEAN PARLIAMENT AND OF THE COUNCIL of 22 September 2010 on the protection of animals used for scientific purposes. The protocol was approved by the "Comité d’Ethique en Expérimentation Animale de l’Université Claude Bernard Lyon 1" (CEEA-55; DR2014-09). The capture of roe deers was carried out only by competent persons using methods which do not cause the animals avoidable pain, suffering, distress or lasting harm.

### Ticks collection, protein and DNA extraction

One hundred and twenty semi-engorged *I*. *ricinus* ticks were collected from roe-deer (*Capreolus capreolus*) in the Chize forest (Northern France) in February 2014. semi-engorged ticks were selected as this stage presents the highest combined development of the two investigated organs, ovaries and salivary glands, and also presents a high concentration of *M*. *mitochondrii* symbionts [8]. Ticks were manually dissected under a stereomicroscope Leica (Wetzlar, Germany), to collect salivary glands and ovaries. Salivary glands and ovaries from twenty ticks were pooled in 100 μL PBS with 1.5 μL of 1x protease inhibitor (Sigma). After mechanical disruption of tissues, 20 μL of lysate were recovered for subsequent DNA extraction. The remaining volume was subjected to sonication with Digital Sonifier 450 (Branson Ultrasonic Corporation, Danbury, CT, USA), with three five-second treatments. Each sample was then centrifuged at maximum speed for 10 min and supernatants were recovered and stored at -80°C until use. DNA from each sample was extracted using the Qiagen DNeasy Blood and Tissue Kit (Hilden, Germany) following manufacturer instructions. DNAs were eluted in 50 μL of sterile water and stored at -20°C until molecular analysis.

### PCR

The presence of common tick-borne pathogenic bacteria [14] in the extracted DNA was screened using previously described PCR protocols for *Borrelia burgdorferi* [15], *Anaplasma* spp., *Ehrlichia* spp. and *Rickettsia* spp. [16].Samples negative for the presence of pathogens were subsequently analyzed for absolute quantification of *M*. *mitochondrii* content using a previously described Sybr green real-time PCR approach [8] based on the amplifications of a fragment of the *M*. *mitochondrii gyrB* gene (coding for the protein gyrase B) and a fragment of the *I*. *ricinus* nuclear gene *cal* (coding for the protein calreticulin). Results were expressed as ratio of *gyrB*/*cal* copy numbers.

### Quantification of proteins

The Bicinchoninic Acid **(**BCA) assay [17] was applied to obtain the exact quantification of each pool of proteins extracted from salivary glands and ovaries. Bovine serum albumin was the standard protein used for the production of calibration curves, in the range of concentrations between 5 and 25 μg/mL.

### Two-Dimensional Electrophoresis (2-DE)

About 250 μg of extracted proteins were dissolved in 125 μL of rehydration buffer (8 M urea, 4% CHAPS (w/v), 65 mM DTE, 0.8% carrier ampholytes (v/v), 0.5% bromophenol blue) and loaded onto 7 cm IPG strips, with nonlinear (NL) pH 3–10 or linear pH 4–7 gradient range, Amersham Biosciences (Amersham, UK). Strips were rehydrated without applying voltage for 1 h at 20°C. The first-dimensional IEF was carried out at 15°C using an Ettan IPGphor system (Amersham Biosciences), programmed with the following voltage gradient: 30 V for 8 h, 120 V for 1 h, 500 V for 0.5 h, 1000 V for 0.5 h and 5000 V until a total of 25–27 kV/h was reached. Reduction/alkylation steps were applied between the first and the second dimension. The focused IPG strips were incubated for 15 min at room temperature in 6 M urea, 2% (w/v) SDS, 50 mM Tris pH 6.8, glycerol 30%, containing 2% (w/v) DTE, followed by a second incubation of 15 min in the same buffer containing 2.5% (w/v) iodoacetamide and 0.5% bromophenol blue. At the end of the IEF step, strips were hold in place with 0.4% low melting temperature agarose and loaded onto 8x6 cm slabs, 12.5% SDS polyacrylamide gels. Electrophoresis was carried out at a constant current of 10 mA per gel in a PROTEAN II xi 2-D Cell equipment Bio-Rad (Berkeley, California), until the buffer front line was 1 mm from the bottom of the gels. The 2-DE gels were stained with ‘‘Blue silver” (colloidal Coomassie G-250 staining), according to Candiano et al [18]. Digital images of stained gels were acquired using VersaDoc Imaging Model 3000 (BioRad) and then subjected to quali/quantitative analysis using the PD Quest (BioRad) version 8.0.1 software. Scanned images were filtered and smoothed to remove background noise, vertical/horizontal streaking and gel artifacts and then normalized to eliminate the variability of each sample. The software then determined the amount of spots present and calculated their intensity by applying the following algorithm: peak value (ODs/image units) *σ_x_*σ_y_ (standard deviations in x and y).

### Reproducibility of the study

To verify the reproducibility of the study, 2-DE maps were obtained in triplicate for each of the analyzed salivary glands (SG) and ovary tissues (OT) pools. Those presented in this report are the best representative gels among all generated that showed spots consistently present. Experimental steps concerning sample preparation, electrophoretic run and gel staining were performed ‘‘in parallel” on all samples.

### *In situ* enzymatic digestion

Enzymatic digestion was performed as previously described [19]. Briefly, the selected spots were carefully excised from the gel, placed into Eppendorf tubes and broken into small pieces. This material was then washed twice with aliquots (200 μL) of 100 mM ammonium bicarbonate buffer pH 7.8, 50% acetonitrile (ACN) and kept under stirring overnight, until complete destaining. Gels were dehydrated by addition of ACN (100 μL). After removal of the organic solvent, reduction was performed by addition of 50 μL of 10 mM Dithiothreitol (DTT) solution (40 min at 37°C). DTT was replaced with 50 μL of 55 mM iodoacetamide for 45 min at 56°C. This solution was removed and the gel pieces were washed twice with 200 μL of 100 mM ammonium bicarbonate for 10 min, while vortexing. The wash solution was removed and gel dehydrated by addition of 200 μL of ACN until the gel pieces became an opaque-white color. ACN was finally removed and gel pieces were dried under vacuum. Gels were rehydrated by addition of 75 μL of 100 mM ammonium bicarbonate buffer pH 7.8, containing 20 ng/μL sequencing grade trypsin (Promega, Madison, WI, USA) and digestion was performed incubating overnight at 37°C. Following enzymatic digestion, the resultant peptides were extracted sequentially from gel matrix by a three-step treatment (each step at 37°C for 15 min) with 50 μL of 50% ACN in water, 5% trifluoroacetic acid (TFA) and finally with 50 μL of 100% ACN. Each extraction involved 10 min of stirring followed by centrifugation and removal of the supernatant. The original supernatant and those obtained from sequential extractions were pooled, dried and stored at -80°C until mass spectrometric analysis. At the moment of use, the peptide mixture was solubilized in 100 μL of 0.1% formic acid (FA) for MS analyses.

### LC-MS/MS

All analyses were carried out on an LC-MS (Thermo Finnigan, San Jose, CA, USA) system consisting of a thermostated column oven Surveyor autosampler controlled at 25°C, a quaternary gradient Surveyor MS pump equipped with a diode array detector, and an Linear Trap Quadrupole (LTQ) mass spectrometer with electrospray ionization ion source controlled by Xcalibur software 1.4. Analytes were separated by RP-HPLC on a Jupiter (Phenomenex, Torrance, CA, USA) C_18_ column (150 x 2 mm, 4 μm, 90 Å particle size) using a linear gradient (2–60% solvent B in 60 min) in which solvent A consisted of 0.1% aqueous FA and solvent B consisted of ACN containing 0.1% FA. Flow-rate was 0.2 mL/min. Mass spectra were generated in positive ion mode under constant instrumental conditions: source voltage 5.0 kV, capillary voltage 46 V, sheath gas flow 40 (arbitrary units), auxiliary gas flow 10 (arbitrary units), sweep gas flow 1 (arbitrary units), capillary temperature 200°C, tube lens voltage –105 V. MS/MS spectra, obtained by CID studies in the linear ion trap, were performed with an isolation width of 3 Th *m/z*, the activation amplitude was 35% of ejection RF amplitude that corresponds to 1.58 V.

Data processing was performed using Peaks studio 4.5 software. An ad-hoc database was obtained selecting from the NCBI database all the protein sequences belonging to the following taxonomic groups: *Ixodida* (taxid:6935), *Cervidae* (taxid:9850), *Borrelia* (taxid:138), *Rickettsiales* (taxid:766). The mass lists were searched against the SwissProt and the ad-hoc protein database under continued mode (MS plus MS/MS), with the following parameters: trypsin specificity, five missed cleavages, peptide tolerance at 0.2 Da, MS/MS tolerance at 0.25 Da, peptide charge 1, 2, 3+, and experimental mass values: monoisotopic.

### Western Blotting

Western blot analysis was effected starting from 100 micrograms of proteins extracted from the OT and SG pool that exhibited the highest concentrations of *M*. *mitochondrii* based on *GyrB/cal* gene ratio. Separated proteins were transferred onto nitrocellulose membrane by using a Trans Blot Electrophoresis Transfer Cell (BioRad) and applying a current of 200 mA for 1.20 h in running buffer (25 mM Tris pH 8.3, 192 mM glycine, 20% methanol). To verify the transfer of proteins, the membrane was stained with Ponceau Red and washed with PBS (10 ml) for 10 min. After 1h incubation in 5% milk (10 ml) diluted in PBS and three additional washes with PBST(0,1% Tween (10 ml), the membrane was incubated overnight with th polyclonal antibodies against the *M*. *mitochondrii* flagellar protein FliD [10] at a dilution 1:5000 in 1% milk. After washing the membrane three times with PBST (10 ml), incubation with the secondary antibody (Dako, Glostrup, Denmark) was carried out for 1 h at room temperature with polyclonal goat anti-rabbit immunoglobulin diluted 1:2000 in 1% milk in PBST. The membrane was finally washed three times with PBS and incubated in ECL Prime solution (GE Healthcare, Uppsala, Sweden). Immunoblots were acquired with the ImageQuant LAS 4000 analyzer (GE Healthcare).

## Results

### PCR

Concentration of *M*. *mitochondrii* and presence of tick-borne pathogens was assessed in all the OT and SG pools by performing PCR on the DNA extracted from the samples. Two out of six OT and SG pools were positive to tick-borne pathogens and were thus excluded from subsequent analyses. PCR for the detection of *M*. *mitochondrii* was performed on the remaining samples and all OT and SG pools resulted, as expected, positive to *M*. *mitochondrii*. The copy numbers of *gyrB* and *cal* genes and the *gyrB/cal* x1000 ratios are provided in S1 Table of Supporting Information.

### Two-dimensional electrophoresis with nonlinear pH 3–10 gradient range

To identify *I*. *ricinus* proteins differentially expressed between OT and SG, as well as proteins of the *M*. *mitochondrii* symbiont, parallel 2-DE analyses were performed on the four salivary glands (SG) and ovary tissues (OT) of *I*. *ricinus* adult ticks that were infected by *M*. *mitochondrii* that resulted free from other bacterial pathogen presence. Gels were scanned and spots were detected using the spot detection wizard tool, after defining and saving a set of detection parameters. Following spot detection, the original gel scans were filtered and smoothed to clarify spots, remove vertical and horizontal streaks, and remove speckles. Three dimensional Gaussian spots were then created from filtered images. Three images were created from the process: the original raw 2-D scan, the filtered image, and the Gaussian image. A match set for each pool was then created for comparison after the gel images had been aligned and automatically overlaid. If a spot was saturated, irregularly shaped, or otherwise of poor quality, then the Gaussian modeling was unable to accurately determine quantity. In these cases, the spot was defined in the filtered image using the spot boundary tools. Thus, for each pool, a master gel was produced which included protein spots only if present at least in two out of the three gels. The mean spot number in Coomassie stained gels was 235±29 in SG and 221±21 in OT. A set of spots chosen from the two master gels were excised, destained, digested with trypsin, and peptides were submitted to LC-MS/MS. The MS fragmentation data were searched against the SwissProt and the ad-hoc designed protein databases, and the queries were performed using the Peaks studio 4.5 software. A total of 47 proteins were identified, 20 from SG and 27 from OT. A complete list of the identified proteins is presented in S2 Table.

**Fig 1:**
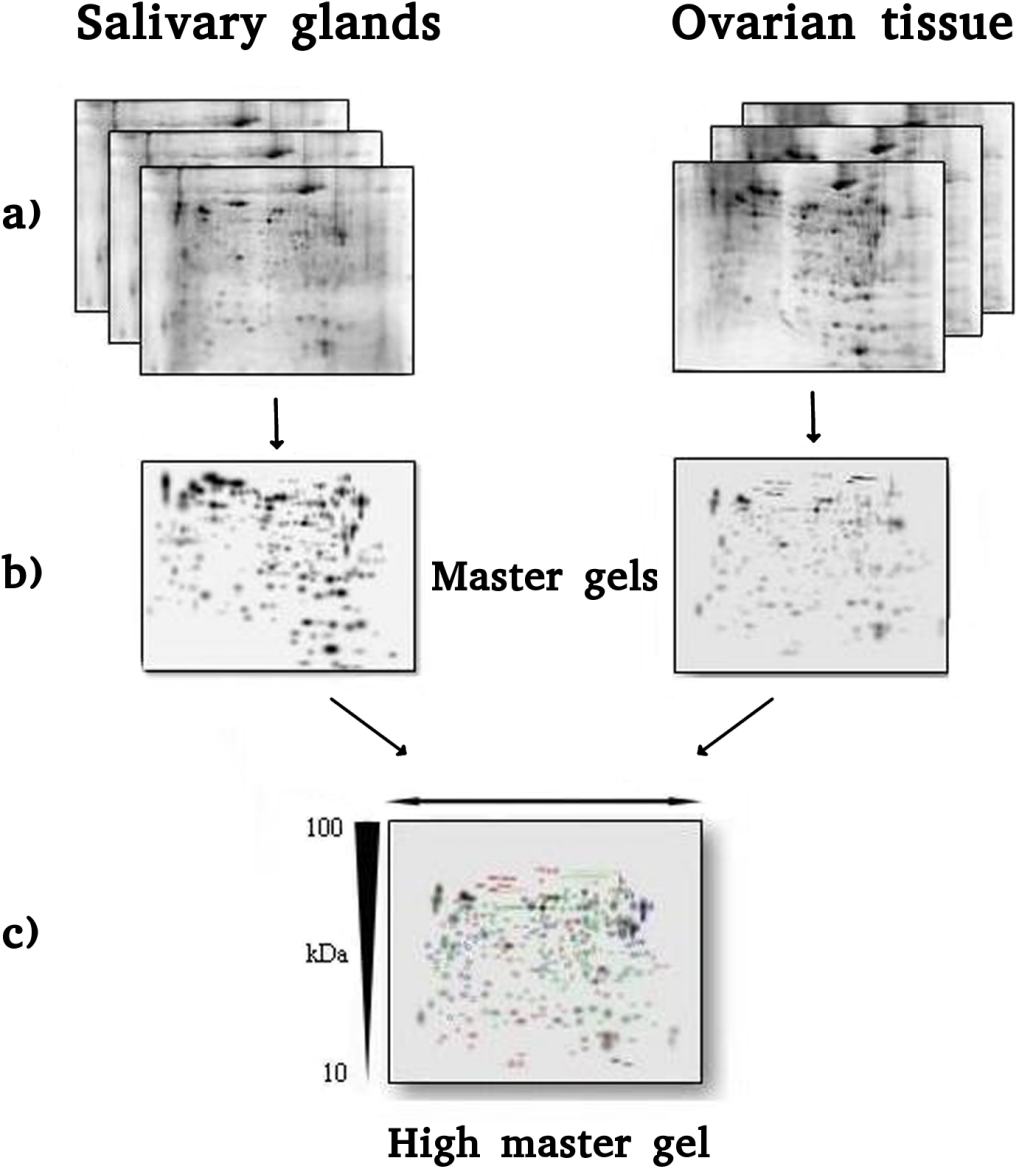
(A) 2-DE maps of three different pools of salivary gland (SG, left) and ovarian tissue (OT, right) of *I*. *ricinus*, obtained by performing IEF on 7 cm IPG strips with 3–10 NL pH range and SDS-PAGE in the second dimension on 8x6 cm slabs, 12.5% T gels. (B) SG and OT master gels obtained merging the three gels for each sample type. (C) 2-DE High Master Gel created comparing the SG and OT gels.

The master gels from both SG and OT pools showed similar patterns of proteins such that they could be matched to each other. This facilitated the correlation of gels and the creation of a virtual image, indicated as high master gel (HMG), comprehensive of all matched spots derived from master gels. The procedure described is summarized in Fig 1.

### Differentially expressed proteins

**Fig 2:**
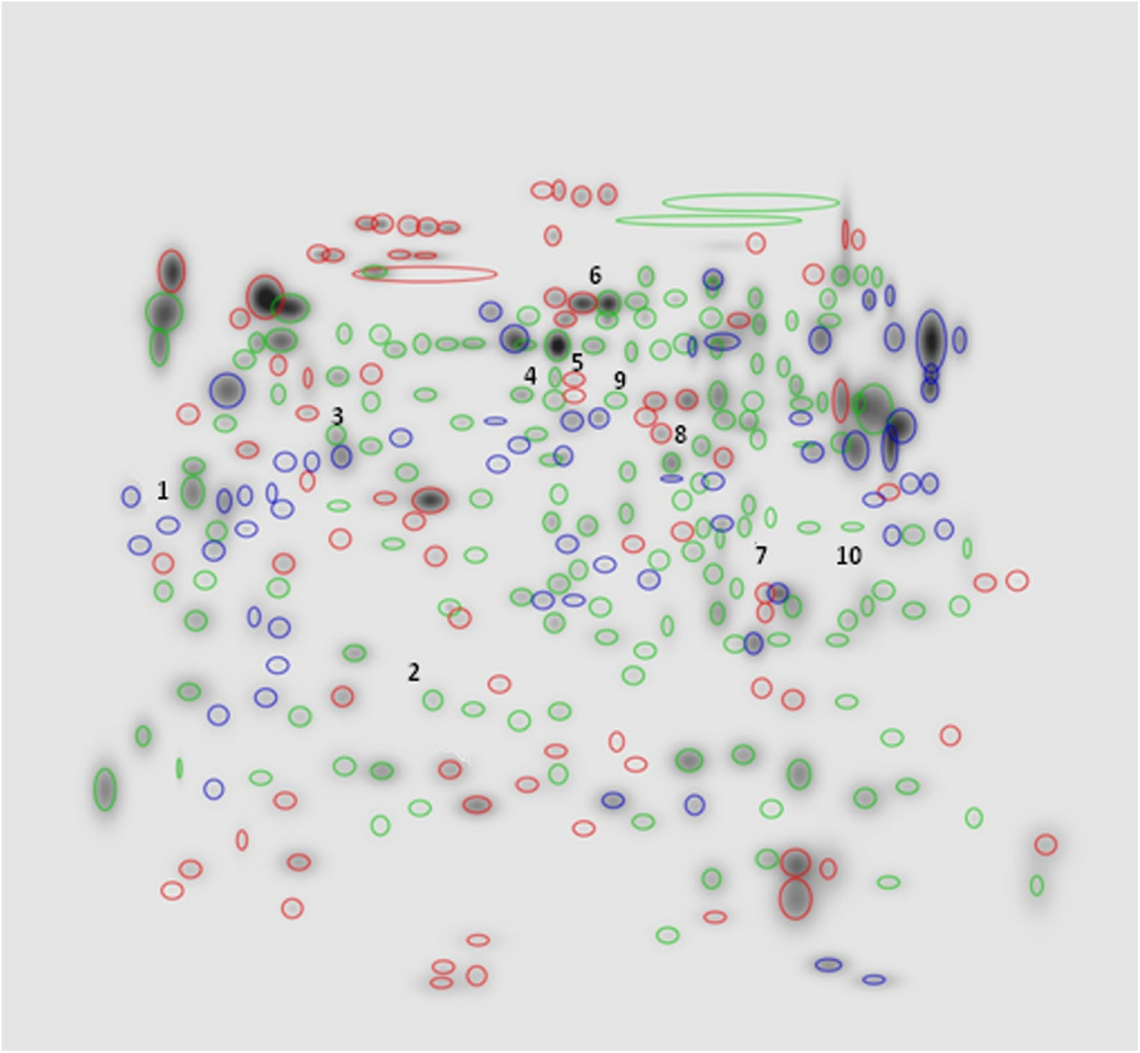
High Master gel, showing qualitative differences between the SG and OT 2-DE master gel patterns (NL, pH 3–10 gradient range). Labelled in green: spots (n = 170 ± 25) common to both SG and OT. Labelled in red: spots (n = 81) exclusive of SG. Labelled in blue: spots (n = 57) detected solely in OT.

Comparison of 2-DE patterns for SG and OT revealed several qualitative and quantitative differences between the two sets of pools. In terms of presence/absence of spots, qualitative differences are represented in Fig 2. As shown, while the majority of spots were common to both SG and OT (170 ± 25, evidenced in green), some protein spots present in SG profile were absent from the OT one and viceversa. In particular, 81 spots (marked in red) were exclusive of SG and 57 spots (labelled in blue) were detected solely in OT.

**Fig 3:**
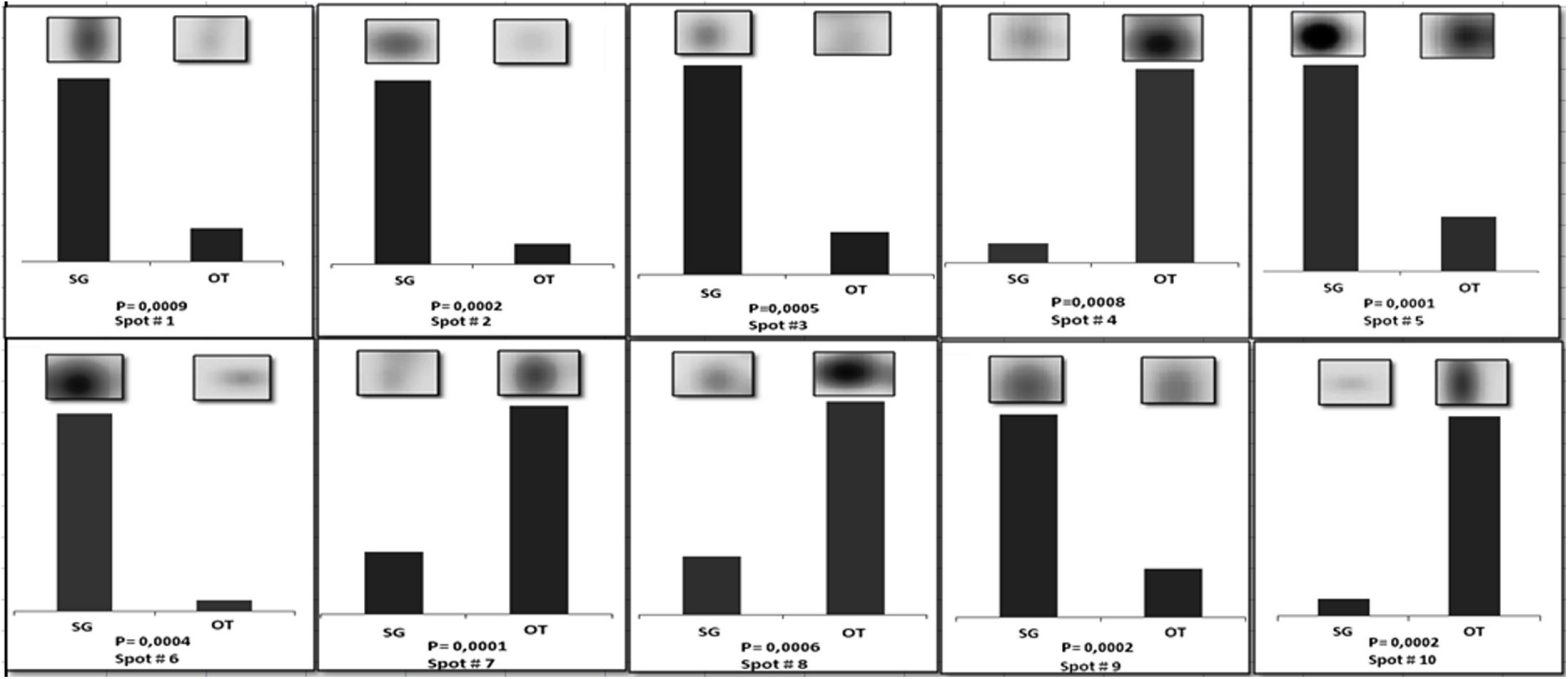
Set of panels showing the density variances between SG and OT pools for spots 1 to 10. In each panel the region of the stained gel containing the spot of interest was magnified (inset) and the up-/downregulation graphically represented. Pvalue indicating statistical significant density variance (T-test) is reported in each panel.

Spot quantities of all gels were normalized to remove non expression-related variations in spot intensity, and data were exported as clipboard for further statistical analysis. The raw amount of each protein in a gel was divided by the total quantity of all proteins (spots) that were included in that gel. The results were evaluated in terms of spot optical density (OD). Statistical analysis of PDQuest data allowed to assess differences in protein abundance on a protein-by-protein basis. According to guidelines for differential proteomic research, only spots that showed a change in density at *p* < 0.01 (by Student's t-test) were considered “differentially expressed” in the two pools of samples. This term was used here meaning differential protein abundance determined by several processes, including changes in protein biosynthesis and modification or degradation. Using these criteria, 21 spots differed by the ratio indicated above and were selected by the statistical program as spots having significant differences in the relative abundance between SG and OT. In particular, ten among these spots (indicated by numbers 1 to 10 in HMG of Fig 2) showed 4- to 18-fold increase/decrease in density. A set of panels, shown in Fig 3, was generated to highlight density variances of these spots between the two sets of pools (i.e. SG and OT).

In these panels the region of stained gel containing the spot of interest was zoomed and up-/downregulation graphically represented. Efforts have been devoted to the identification of these proteins, to investigate whether they might have an involvement in the biological processes characteristic of ovaries and salivary glands, or in the interaction between *M*. *mitochondrii* and *I*. *ricinus*.

These spots were thus carefully excised from the gel, destained, digested with trypsin, and peptides were submitted to LC-MS/MS following the procedure detailed in the Materials and Methods section. The MS fragmentation data were searched against the SwissProt and the ad-hoc designed protein databases, and the queries were performed using the Peaks studio 4.5 software. All but two (spots 2 and 9) of the queried proteins were identified. The low abundance of proteins corresponding to spots 2 and 9 most likely determined the poor quality of their MS signals and failure in their identification. The fact that unique proteins were identified for all other analyzed spots suggested that, at least for these spots, spot overlap was minimized.

Detailed identification data, including accession number, theoretical p*I*, molecular mass, percent of sequence coverage, number of peptides identified, and MOWSE score of each of the nine proteins identified are reported in Table 1.

**Table 1:** Up and downregulated proteins identified by LC-MS/MS.

Spot	Accession	Description	Mass	Score (%)	Coverage (%)	Query matched
1	gi|442756551|gb|JAA70434.1|	Putative heat shock 70 kda protein 5 [Ixodes ricinus]	72,595	99	8,05%	5
2	/	Not detected	/	/	/	/
3	gi|322422107|gb|ADX01224.1|	Beta actin [Ixodes ricinus]	16,038	90	6,94%	1
4	gi|215497327|gb|EEC06821.1|	Enolase, putative [Ixodes scapularis]	21,493	90	4,52%	1
5	gi|442753241|gb|JAA68780.1|	Putative enolase [Ixodes ricinus]	47,145	99	23,79%	6
6	gi|215491972|gb|EEC01613.1|	Protein disulfide isomerase, putative [Ixodes scapularis]	54,929	98	6,38%	4
7	gi|442748259|gb|JAA66289.1|	Putative 3-hydroxy-3-methylglutaryl-coa reductase [Borrelia spp]	10,741	20	22,43%	2
8	gi|597718071|gb|AHN19768.1|	Serum albumin, partial [Cervus nippon]	66,15	75	6,67%	4
9	/	Not detected	/	/	/	/
10	gi|442754645|gb|JAA69482.1|	Putative heat shock protein [Ixodes ricinus]	36,782	82	4,01%	2

Additional information concerning the primary sequence of all peptides identified for each protein analyzed was included in S3 Table of Supporting Information.

### Western blotting

**Fig 4:**
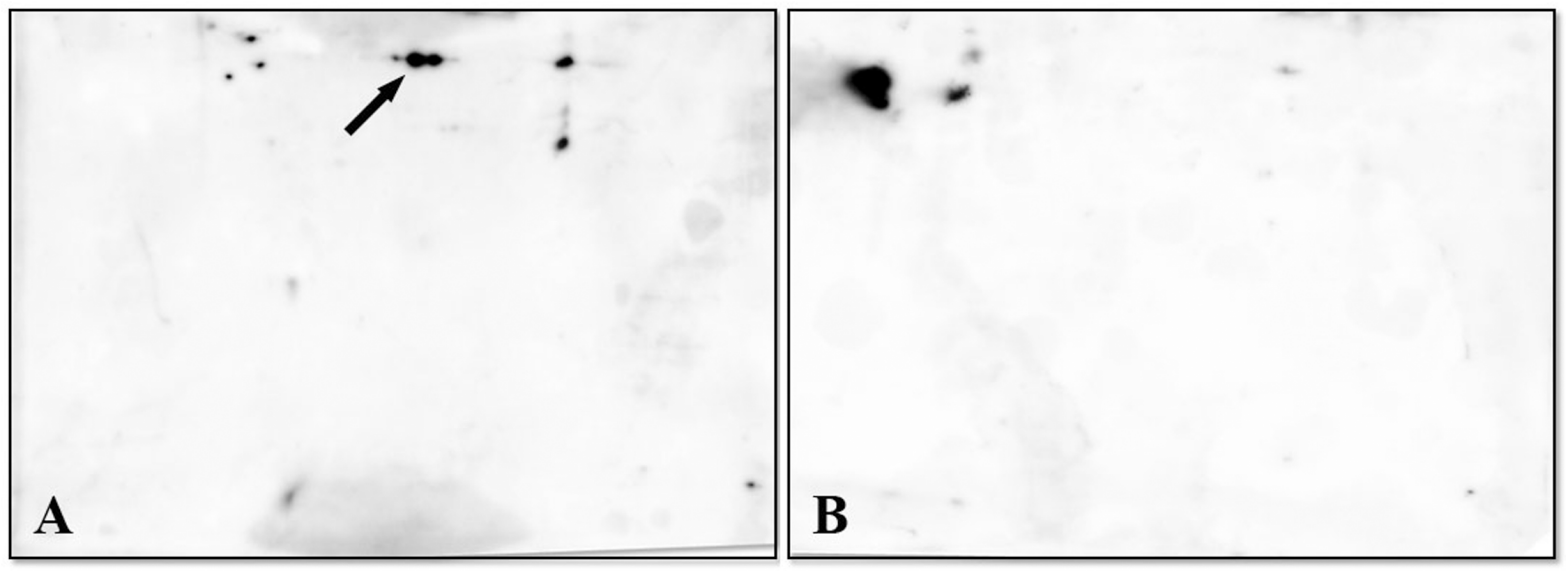
Immunoblotting of proteins from SG and OT profiles generated as indicated in Fig 1. PVDF membranes were incubated with the rabbit polyclonal antibodies anti-FliD of *M*. *mitochondrii*, followed by anti-rabbit antibody. The protein spot(s) indicated by an arrow in Panel A (OT pool) was tentatively assigned to FliD. Panel B shows the SG profile in which the hypothetical FliD spot is undetectable.

Given the aim of our study, proteins from SG and OT profiles of the pool that exhibited the highest concentrations of *M*. *mitochondri* were transferred onto PVDF membranes and incubated with the polyclonal anti-FliD antibodies, followed by anti-rabbit antibody. Based on: i) its position (pI/Mr) on the PVDF membrane and ii) its recognition by the antibody, the protein spot indicated by an arrow in panel A (OT pool) of Fig 4, was tentatively assigned to FliD. As shown in panel B of Fig 4, despite the appearance of interfering spots, the hypothetical FliD spot was undetectable in the SG profile. This result is expected and the load of *M*. *mitochondrii* bacteria is much higher in ovaries than in salivary glands (S1 Table) [10, 11].

To achieve identification, the immunoreactive protein spot was thus excised from the original OT gel and submitted to the LC-MS/MS procedure indicated above. The results (shown in Table 2) confirmed what appeared evident from the visual inspection of the gel, i.e. the analyzed spot, rather than comprising a single polypeptide chain, was a mixture of at least three components, i.e. Endoplasmic Reticulum Protein 60, putative actin 2, and an unknown protein from *Borrelia*. Information concerning the primary sequence of all peptides identified for each protein analyzed have been included in S4 Table of Supporting Information.

**Table 2:** List of proteins identified under the immunoreactive spot.

Accession	Description	Mass	Score (%)	Coverage(%)	Query matched
gi|442747467|gb|JAA65893.1|	Putative erp60 [Ixodes ricinus]	52,115	98	6,45%	3
gi|556065071|gb|JAB75571.1|	putative actin-2 [Ixodes ricinus]	36,27	89	9,79%	2
gi|6841058|gb|AAF28881.1|	unknown [Borrelia hermsii]	29,518	25	3,92%	1

We hypothesized that the fact that the putative flagellar protein FliD was, most likely, less abundant compared to the bulk of other proteins present in the spot, prevented its identification. This hypothesis was strengthened by the poor quality of MS sequence data obtained from third spot (or cluster of spots). This indeed made it difficult to define exactly whether the FliD protein of was actually present within the spot(s) considered.

### Two-dimensional electrophoresis with pH 4–7 gradient range: identification of FliD

**Fig 5:**
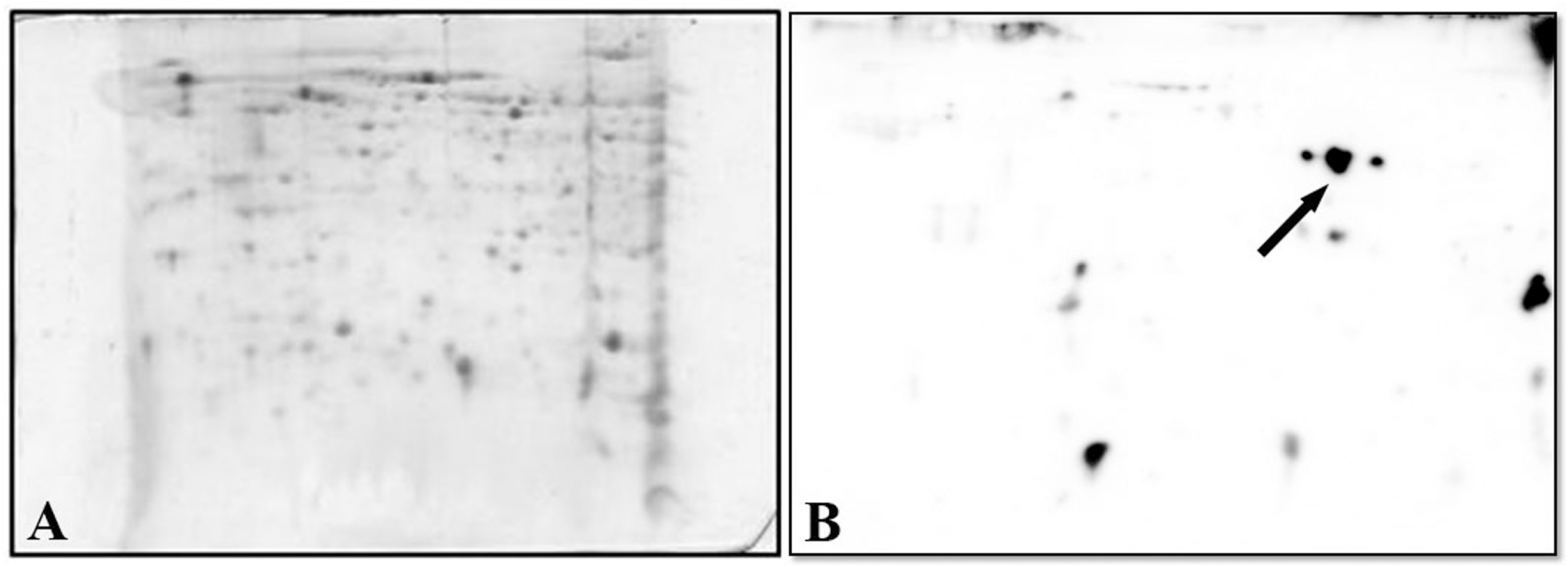
(A) 2-DE map of OT obtained by performing IEF on a 4–7 linear pH range and SDS-PAGE on a constant 12,5% T in the second dimension, to separate proteins clustered in the single spot shown in Fig 4. (B) Immunoblotting of the gel slab indicated in Panel A. Arrow points to spot originated from separation and identified as FliD.

In an effort to overcome the limitations indicated above and to definitively establish (or exclude) the presence of FliD under the spot(s) examined in the OT pool, we worked on the optimization of the electrophoretic conditions. After performing a extensive set of trials with various electrophoretic conditions, the best option was found to be the application of a narrow range pH gradient (linear pH 4–7). This provided a better resolution of proteins, minimizing potential spot overlaps (Fig 5A). The immunoreactive protein spot (indicated by an arrow in PVDF membrane, B) was evidenced in OT profile obtained under the experimental conditions mentioned above.

As a validation of data discussed above, no spot was reactive against the antibody in SG profile (data not shown). After spot excision and tryptic digestion, LC-MS identification confirmed the presence of flagellar protein FliD under this spot (Table 3).

**Table 3:** List of proteins that confirmed the presence of FliD.

Accession	Description	Mass	Score (%)	Coverage(%)	Query matched
/	Flagellar protein FLID OS = Midichloria mitochondrii	100,58	84	14,64%	13
gi|442747467|gb|JAA65893.1|	Putative erp60 [Ixodes ricinus]	52,115	98	6,45%	3

The primary sequence of peptides found for identification of protein analyzed was included in S5 Table of Supporting Information.

## Discussion

### Analysis of common proteins differentially expressed

A recent study presented the identification of hundreds of proteins in salivary glands of *I*. *ricinus* in the presence of the pathogenic tick-borne spirochete *B*. *burgdorferi*, demonstrating that the expression of proteins modulated by infection differed as a function of the various strains of *B*. *burgdorferi* [20]. In the present study, a total of 47 spots, 27 from OT and 20 from SG were selected for sequencing. Our results were fully congruent with those previously published, validating our approach and confirming the high expression level of a number of proteins, such as Heat shock protein, Protein disulfide isomerase, Enolase, Actin, Hemelipoglycoprotein precursor (putative) etc. This sequencing effort detected only proteins that could be readily identified as belonging to the tick proteome, or that could not be identified unambiguously, but did not reveal the presence of proteins belonging to the bacterial symbiont *M*. *mitochondrii*. To evaluate whether detection of symbiont proteins was possible, we thus designed a specific immunoproteomic approach, described above and discussed below.

To explore qualitative and quantitative differences between SG and OT of *I*. *ricinus* infected by *M*. *mitocondrii*, and to identify tissue-specific proteins, as well as proteins involved in the interaction between the bacterium and the tick, we compared the proteomic profiles of these tissues. The reproducible patterns generated for both tissues evidenced 170 ± 25 spots shared between SG and OT. In addition, 81 protein spots were exclusive of SG profile and 57 spots were detected solely in OT. In particular, we selected and investigated the 10 proteins that exhibited the largest changes in density (4- to 18-fold increase/decrease, see Fig 2, Fig 3 and Table 1). Proteins under spots 1 and 10, both identified as putative heat shock proteins (HSP), were heavily differentially expressed. Putative HSP70 detected under spot 1 was 6-fold more abundant in SG than in OT. By contrast, the putative HSP identified under spot 10 was 11-fold more abundant in OT than in SG. Heat shock proteins are chaperones that, together with other stress response proteins, are well known to protect cells and organisms from environmental stress. HSP70, is involved in many cellular processes, including folding and refolding of nascent and/or misfolded proteins, protein translocation across membranes, and degradation of terminally misfolded or aggregated proteins [21]. The role played by HSP proteins in the growth and survival of *I*. *ricinus* is potentially very important. Being involved in the binding and presentation of antigens to the immune system, they constitute candidate molecules that could be involved in tick immune response to pathogen infection. Interestingly, a connection may exist between pathogen infection and tick response to stress conditions. In response to heat and other stress (cold, hunger), nearly all ticks undergo diapause. Indeed HSPs involved in the diapause of multiple species of insects were reported [22], suggesting that they may play key roles in the physiological response to stress of other arthropods, such as ticks. HSP70 is more expressed in salivary glands than in ovarian tissue and midgut and its expression increases with female tick feeding, suggesting a possible role of this protein during blood ingestion and/or digestion [23]. We speculated that this could be ascribed to the great changes in structure that the salivary glands of hard ticks undergo during blood feeding with an increase in size and the acceleration of protein synthesis [24]. However, the fact that HSP70 was found to be down regulated in *Anaplasma phagocytophilum* infected whole *Ixodes scapularis* ticks, guts and salivary glands [23], may suggest that these proteins have a different function during pathogen infection.

The protein identified under spot 3 was β-actin. The reason of its 5-fold higher expression in SG compared to OT is still a matter of speculation. First, we hypothesized that this was a consequence of the importance of SG in tick feeding. Actin is an important structural protein required for exoskeleton rearrangement during tick engorgement [25] and is a common target of many bacterial proteins. It has been shown that the cellular responses induced by a variety of stimuli and pathogens involve changes in cell morphology and the polymerization state of actin [26–27–28]. Studies in prokaryotes and eukaryotes demonstrated that nutrition and stress affect the expression of housekeeping genes [29]. For example the low expression of actin shown in the unfed first instars nymphs of *I*. *scapularis* is likely due to low nutrition levels since they have not yet taken the blood meal and the nutrients incorporated into the eggs have been depleted by larval development [30]. The significant differences observed during and immediately after feeding in females are likely related to the dynamic changes that occur in the physiology of ticks preparing for reproduction. It has also been shown that silencing the expression of actin in the soft tick *Ornithodoros moubata* resulted in impairment of tick feeding by a global attenuation of tick activity unrelated to specific function associated with engorgement [29]. Finally, further aspects should be taken into consideration. *I*. *ricinus*, as *I*. *scapularis*, are vectors of bacterial pathogens including *A*. *phagocytophilum*, and *B*. *burgdorferi* [30,31]. To persist in their hosts, obligate intracellular bacteria have evolved a variety of mechanisms including modulating host signaling and the actin cytoskeleton [32]. If this hypothesis proves correct, it may be speculated that the high concentration of actin detected in SG of *I*. *ricinus* could be the result of a sort of survival strategy developed by the symbiont *M*. *mitochondrii* to persist in its arthropod vector.

The identification of enolase under spots 4 and 5 attracted our interest. Alpha-enolase, one of the most abundantly expressed proteins in human cytosol, is a key glycolytic enzyme that converts 2-phosphoglycerate to phosphoenolpyruvate [33]. In blood-feeding arthropods this protein is secreted in saliva and inoculated into the host during feeding. The finding of 4-fold higher expression of enolase in SG compared to OT (spot 5) was not surprising. This result may account for one of the pivotal roles of this enzyme. Enolase, in fact, promotes fibrinolysis and maintains blood fluidity during blood ingestion and distribution in the tick midgut. Fibrinolysis is the natural process of fibrin clot solubilization and, in ticks, this process is essential for dissolving any clot that might be formed during feeding, as well as preventing clotting of the ingested blood meal in its midgut [34]. This said, the higher expression level in OT (10-fold more expressed than in SG) of another putative enolase (spot 4) was a result apparently in contradiction with the previous one. However, the multifunctionality of this protein in both prokaryotes and eukaryotes may probably account for this finding. In fact, it has been shown in *Rhipicephalus microplus* [35] that, to support the energy-intensive processes of embryogenesis, before blastoderm formation, glycogen reserves are preferentially mobilized. As a consequence, protein degradation and gluconeogenesis intensify to supply the embryo with sufficient glucose to allow glycogen re-synthesis. If glycogen is the main energy source during the early stages of *R*. *microplus* embryogenesis, protein degradation increases during late embryogenesis [35]. Thus, the use of amino acids as a substrate for gluconeogenesis and the subsequent glycogen re-synthesis play an important role during the stages of *R*. *microplus* embryogenesis. Protein metabolism depends strongly on the substantial expression and activity of carbohydrate metabolism enzymes and alpha-enolase is a key glycolitic enzyme [36].

The protein identified under spot 6 was disulfide isomerase (PDI), a 55 KDa multifunctional protein that participates in protein folding, assembly, and post-translational modification in the endoplasmatic reticulum [37]. The fact that it was 18-fold-more expressed in SG than in OT was not surprising. This protein, together with other saliva enzymes which are putatively associated with antioxidant functions (i.e. glutathion-S-transferase, cytochrome c oxidase, oxidoreductase, NADH dehydrogenase), plays an important role in oxidative stress [38]. Tick-feeding, in fact, induces injuries and oxidative stress leading to production of reactive oxygen and nitrogen species (ROS and RNS) as part of the wound healing mechanism and anti-microbial defenses. Several lines of research have shown that many parasites including ticks are susceptible to ROS and RNS, as revealed by high expression of anti-oxidant enzymes in these parasites or improved survival of these parasites when anti-oxidant system of their hosts are impaired [38]. The production of antioxidant enzymes can be considered an evasion mechanism of the immune response used by tick for improving the feeding efficiency, and collaterally enhancing transmission of tick-borne diseases. It is also interesting to note that, given that the tissue destroying effects of oxidative stress products are non-selective, there is a possibility that tick saliva anti-oxidants are protective to host tissue [38].

### Identification of FLID

One of the goals of this study was to evaluate whether it is possible to detect proteins from the bacterial symbiont *M*. *mitochondrii* starting from protein extracts of ovaries and salivary glands of the hard tick *I*. *ricinus*. Due to the higher symbiont load in OT we expected this to be easier in this tissue, however none of the 2DE gel spots identified resulted to be of *M*. *mitochondrii*, neither from the OT, nor from the SG. We thus questioned whether this was a result of the low abundance of symbiont proteins or to some kind of technical issue. To investigate this, we performed an immunoproteomic approach based on the detection of a single *M*. *mitochondrii* protein (FliD) in a blotting experiment after narrow range pH gradient (linear pH 4–7) 2DE separation of OT and SG (see materials and methods). As shown in Fig 5, a spot potentially corresponding to FliD, based on the chemical properties of the protein, was detected in OT blotting, but not in SG blotting. When analyzing the corresponding region in the original 2DE gel, a cluster of spots was detected. Careful excision of these spots followed by LC/MS-MS allowed the identification of erp60, as well as of the expected *M*. *mitochondrii* protein FliD. This result indicates that symbiont proteins can indeed be identified using a proteomic approach, but that this is impaired by low protein quantities and by clustering with *I*. *ricinus* proteins. Specific approaches, such as the use of alternative narrow range pH gradients, immunoproteomic detection are thus needed to investigate the symbiosis between *I*. *ricinus* and *M*. *mitochondrii* from a proteomic point of view.

Here we presented a methodological framework that will pave the way for future studies on the proteomics of *I*. *ricinus*, with the goal of better understanding the biology of this vector and of its symbiont *M*. *mitochondrii*, but also to be the basis of immunoproteomic approaches that could prove useful for detecting novel antigenic proteins for innovative diagnostic and vaccination approaches.

## Supporting Information

S1 Table PCR results on DNA extracted from ovarian tissue and salivary glands pools. GyrB and Cal gene copy numbers, GyrB Cal gene ratio, PCR positivity to *Borrelia burgdorferi*, *Anaplasma* spp., *Ehrlichia* spp. and *Rickettsia* spp. are indicated.
(DOCX)

S2 Table Complete list of the 47 identified proteins, 20 from salivary glands and 27 from ovarian tissue. (DOCX)

S3 Table List of proteins up and downregulated with additional information such as the primary sequence of peptides. (DOCX)

S4 Table List of proteins identified under the immunoreactive spots with additional information such as the primary sequence of peptides. (DOCX)

S5 Table Primary sequence of peptides found for identification of analyzed proteins. (DOCX)
