# Supplementary material for: Ixodes ricinus and Its Endosymbiont Midichloria mitochondrii: A Comparative Proteomic Analysis of Salivary Glands and Ovaries
Source: PLoS One. 2015 Sep 23;10(9):e0138842. doi: 10.1371/journal.pone.0138842 (PMC4580635; doi:10.1371/journal.pone.0138842)
Supplement: S1 Table — GyrB and Cal gene copy numbers, GyrB Cal gene ratio, PCR positivity to Borrelia burgdorferi, Anaplasma spp., Ehrlichia spp. and Rickettsia spp. are indicated. (DOCX) [file pone.0138842.s001.docx]

| Pool code | *GyrB* copies | *cal* copies | *gyrB/cal* ratio x 1000 |
| --- | --- | --- | --- |
| OV 1 | 373899,9594 | 37,66598829 | 9926726,373 |
| SG 1 | 13880,81163 | 34,89770488 | 397757,1499 |
| OV 2 | 1872603,455 | 1500,759257 | 1247770,718 |
| SG 2 | 168,2698052 | 9,937737539 | 16932,40585 |
| OV 3 | 3738999,594 | 1060,761728 | 3524825,128 |
| SG 3 | 2437,79344 | 487,6015747 | 4999,560228 |
| OV 4 | 1265223,304 | 328,3030099 | 3853827,916 |
| SG 4 | 116,9799564 | 88,44119687 | 1322,686265 |
