# Supplementary material for: Ixodes ricinus and Its Endosymbiont Midichloria mitochondrii: A Comparative Proteomic Analysis of Salivary Glands and Ovaries
Source: PLoS One. 2015 Sep 23;10(9):e0138842. doi: 10.1371/journal.pone.0138842 (PMC4580635; doi:10.1371/journal.pone.0138842)
Supplement: S2 Table — (DOCX) [file pone.0138842.s002.docx]

**Table S2.** Complete list of the 47 identified proteins, 20 from salivary glands (top) and 27 from ovarian tissue (bottom).

| Accession | Mass  kDa | Score (%) | Description | z | Peptides |
| --- | --- | --- | --- | --- | --- |
| gi\|215494047\|gb\|EEC03688.1\| | 71,165 | 99 | heat shock protein, putative [Ixodes scapularis] | 1 | ITITNDK |
|  |  |  |  | 1 | LIGDAAK |
|  |  |  |  | 1 | NTTIPTR |
|  |  |  |  | 1 | STLEPVEK |
|  |  |  |  | 2 | SQVHDIVLVGGSTR |
|  |  |  |  | 1 | DNNLLGK |
|  |  |  |  | 2 | VEIIANDQGNR |
|  |  |  |  | 2 | STAGDTHLGGEDFDNR |
|  |  |  |  | 2 | WLDTNQLADK |
|  |  |  |  | 1 | EIAEAYLGK |
|  |  |  |  | 2 | TTPSYVAFTDTER |
|  |  |  |  | 2 | NALESYSFNIK |
|  |  |  |  | 2 | IINEPTAAAIAYGLDK |
|  |  |  |  | 2 | FEELNADLFR |
|  |  |  |  | 2 | QKELEQVCNPIITK |
|  |  |  |  |  |  |
| gi\|442760941\|gb\|JAA72629.1\| | 34,898 | **99** | Putative carbonic anhydrase, partial [Ixodes ricinus] | 2 | APTDGPILK |
|  |  |  |  | 1 | APTDGPILK |
|  |  |  |  | 2 | QSPIDIVQK |
|  |  |  |  | 2 | NVQSDPFLAENPLR |
|  |  |  |  | 2 | EPIEVSQEQFEAFR |
|  |  |  |  |  |  |
| gi\|442747467\|gb\|JAA65893.1\| | 52,115 | **99** | Putative erp60 [Ixodes ricinus] | 2 | TNDPPVPLIK |
|  |  |  |  | 2 | TLADEDVLVVK |
|  |  |  |  | 2 | MTNDFSVENLEK |
|  |  |  |  | 2 | FLEEYLAGNVK |
|  |  |  |  |  |  |
| gi\|442747295\|gb\|JAA65807.1\| | 54,708 | **99** | Putative erp60 [Ixodes ricinus] | 2 | TNDPPVPLIK |
|  |  |  |  | 2 | MTNDFSVENLEK |
|  |  |  |  | 2 | TLADEDILVVK |
|  |  |  |  | 2 | FLEEYLAGNVK |
|  |  |  |  |  |  |
| gi\|122555\|sp\|P02073.1\|HBB_ALCAA | 16,223 | **99** | RecName: Full=Hemoglobin subunit beta | 1 | MLTAEEK |
|  |  |  |  | 2 | VVTGVANALAHR |
|  |  |  |  | 2 | LHVDPENFR |
|  |  |  |  | 3 | LHVDPENFR |
|  |  |  |  | 2 | VLDAFSEGLK |
|  |  |  |  | 1 | VLDAFSEGLK |
|  |  |  |  | 2 | LLVVYPWTQR |
|  |  |  |  |  |  |
| gi\|122678\|sp\|P21380.1\|HBB_RANTA | 16,167 | **99** | RecName: Full=Hemoglobin subunit beta | 2 | VVTGVANALAHR |
|  |  |  |  | 2 | LHVDPENFR |
|  |  |  |  | 3 | LHVDPENFR |
|  |  |  |  | 2 | AAVTGFWGKVK |
|  |  |  |  | 1 | AAVTGFWGK |
|  |  |  |  | 2 | LLVVYPWTQR |
|  |  |  |  |  |  |
| gi\|556054634\|gb\|JAB70362.1\| | 161,482 | **99** | putative vitellogenin-2 [Ixodes ricinus] | 1 | GSLLSK |
|  |  |  |  | 2 | ENAEGKPLGINR |
|  |  |  |  | 1 | DIALPVYK |
|  |  |  |  | 1 | YSFTK |
|  |  |  |  | 1 | SETAYLR |
|  |  |  |  | 1 | EVYLSAK |
|  |  |  |  | 2 | KFALDQTQDAK |
|  |  |  |  | 2 | GVLSIFQLDLVK |
|  |  |  |  | 2 | FALDQTQDAK |
|  |  |  |  | 2 | LLNQVVGPQPGSTK |
|  |  |  |  | 2 | SQVILSSGYDPK |
|  |  |  |  | 2 | LNNLAVFHEGK |
|  |  |  |  | 2 | YVTTFDLSTDKDK |
|  |  |  |  | 2 | EVEDALPITDR |
|  |  |  |  | 2 | FYYATQNPEWHPR |
|  |  |  |  | 2 | GLHDYWYESDDR |
|  |  |  |  | 3 | VHHIAQSFPQSDEAESLDELK |
|  |  |  |  | 2 | LTDDEAEHFLGK |
|  |  |  |  | 3 | EVEDALPITDRDYDHVYGR |
|  |  |  |  | 1 | FESWGLDK |
|  |  |  |  | 2 | FPEPEWER |
|  |  |  |  | 2 | YDYGGSSVFAQVR |
|  |  |  |  | 2 | TNPDLYLLK |
|  |  |  |  | 3 | TLDLEEVHDANTDTQLPDDLEK |
|  |  |  |  | 2 | TLDLEEVHDANTDTQLPDDLEK |
|  |  |  |  | 1 | IAAFVNVLK |
|  |  |  |  | 3 | FISGLNHLAALEYEDSDIKDVHSK |
|  |  |  |  | 3 | DYFHGYSFETVSLK |
|  |  |  |  | 2 | NYFGEVLGYGLNVK |
|  |  |  |  | 2 | ELQFTLVPAAQDTTTEVEVDLGYK |
|  |  |  |  |  |  |
| gi\|556054820\|gb\|JAB70455.1\| | 175,61 | **99** | putative vitellogenin-2 [Ixodes ricinus] | 1 | GSLLSK |
|  |  |  |  | 2 | ENAEGKPLGINR |
|  |  |  |  | 1 | AFIQTTK |
|  |  |  |  | 1 | DIALPVYK |
|  |  |  |  | 1 | YSFTK |
|  |  |  |  | 1 | SETAYLR |
|  |  |  |  | 1 | EVYLSAK |
|  |  |  |  | 2 | KFALDQTQDAK |
|  |  |  |  | 2 | LHVVSQVPPVEK |
|  |  |  |  | 2 | FALDQTQDAK |
|  |  |  |  | 2 | LLNQVVGPQPGSTK |
|  |  |  |  | 2 | SQVILSSGYDPK |
|  |  |  |  | 2 | LNNLAVFHEGK |
|  |  |  |  | 2 | YVTTFDLSTDKDK |
|  |  |  |  | 2 | EVEDALPITDR |
|  |  |  |  | 2 | FYYATQNPEWHPR |
|  |  |  |  | 2 | GLHDYWYESDDR |
|  |  |  |  | 3 | VHHIAQSFPQSDEAESLDELK |
|  |  |  |  | 2 | LTDDEAEHFLGK |
|  |  |  |  | 3 | EVEDALPITDRDYDHVYGR |
|  |  |  |  | 1 | FESWGLDK |
|  |  |  |  | 2 | FPEPEWER |
|  |  |  |  | 2 | YDYGGSSVFAQVR |
|  |  |  |  | 2 | TNPDLYLLK |
|  |  |  |  | 3 | TLDLEEVHDANTDTQLPDDLEK |
|  |  |  |  | 2 | TLDLEEVHDANTDTQLPDDLEK |
|  |  |  |  | 1 | IAAFVNVLK |
|  |  |  |  | 3 | FISGLNHLAALEYEDSDIKDVHSK |
|  |  |  |  | 3 | DYFHGYSFETVSLK |
|  |  |  |  | 2 | NYFGEVLGYGLNVK |
|  |  |  |  | 2 | ELQFTLVPAAQDTTTEVEVDLGYK |
|  |  |  |  |  |  |
| gi\|215508461\|gb\|EEC17915.1\| | 151,678 | **95** | hemelipoglycoprotein precursor, putative [Ixodes scapularis] | 3 | KKSKNRHR |
|  |  |  |  | 1 | GSLLSK |
|  |  |  |  | 2 | LHVVSQVPPVEK |
|  |  |  |  | 2 | LNNLAVFHEGK |
|  |  |  |  | 2 | SQLLISSGYDPK |
|  |  |  |  | 2 | YDYGGATTFGQIR |
|  |  |  |  |  |  |
| gi\|215504084\|gb\|EEC13578.1\| | 177,654 | **92** | hemelipoglycoprotein precursor, putative [Ixodes scapularis] | 2 | HPELVR |
|  |  |  |  | 1 | GSLLSK |
|  |  |  |  | 1 | DIALPVYK |
|  |  |  |  | 1 | YSFTK |
|  |  |  |  | 3 | EVEDALPVTDREYDHVYGR |
|  |  |  |  | 3 | TLDLEEVHDANTDTQLPEDLER |
|  |  |  |  |  |  |
| gi\|215505979\|gb\|EEC15473.1\| | 56,912 | **99** | protein disulfide isomerase, putative [Ixodes scapularis] | 3 | VDATIETQLAETYEVR |
|  |  |  |  | 1 | TFVDASK |
|  |  |  |  | 2 | GTNEAVEYNGER |
|  |  |  |  | 1 | TLEGLSK |
|  |  |  |  | 2 | RPDLVIAK |
|  |  |  |  | 2 | FDGTANELEHTK |
|  |  |  |  | 2 | QLVDESSDIK |
|  |  |  |  | 2 | AAPEEVTEEEEEEEDKEDK |
|  |  |  |  |  |  |
| gi\|215495481\|gb\|EEC05122.1\| | 60,484 | **99** | chaperonin subunit, putative [Ixodes scapularis] | 2 | VGGSSEVEVNEK |
|  |  |  |  | 2 | VNDALNATR |
|  |  |  |  | 1 | TGVAIVK |
|  |  |  |  | 1 | DGVITVK |
|  |  |  |  | 1 | VGLQVAAVK |
|  |  |  |  |  |  |
| gi\|215495481\|gb\|EEC05122.1\| | 60,484 | **99** | chaperonin subunit, putative [Ixodes scapularis] | 1 | DDTLLLK |
|  |  |  |  | 2 | DDTLLLK |
|  |  |  |  | 1 | LASGVALLK |
|  |  |  |  | 3 | LVQDVANNTNEEAGDGTTTATVLAR |
|  |  |  |  | 2 | VAQIRDEIDLSNSEYEK |
|  |  |  |  | 2 | NVILEQSWGSPK |
|  |  |  |  | 2 | VVEGSDDFGYDALR |
|  |  |  |  | 2 | GYISPYFINTSK |
|  |  |  |  | 2 | AAVEEGIVPGGGTALLR |
|  |  |  |  | 2 | NTYVNMISAGIIDPTK |
|  |  |  |  | 2 | KISNVQTLIPALELANTQR |
|  |  |  |  | 2 | TLSDELEVIEGMK |
|  |  |  |  | 2 | VEFQDALLLFSEK |
|  |  |  |  | 3 | TALLDASGVASLLTTAEAVVVELPKEEK |
|  |  |  |  |  |  |
| gi\|442757975\|gb\|JAA71146.1\| | 47,874 | **99** | Putative protein disulfide-isomerase [Ixodes ricinus] | 2 | VDATVETQLAETYEVR |
|  |  |  |  | 2 | LHNLLFVSK |
|  |  |  |  | 2 | KSPGFEDILK |
|  |  |  |  | 1 | NFDEVVFDK |
|  |  |  |  | 2 | SLMEGAVTSESVQSFVK |
|  |  |  |  | 1 | TFVQDVLDGK |
|  |  |  |  | 2 | TFVQDVLDGK |
|  |  |  |  | 2 | QSLLSQDLPEDWDR |
|  |  |  |  | 1 | ILEFFGLK |
|  |  |  |  |  |  |
| gi\|254590119\|gb\|ACT69481.1\| | 4,326 | **23** | hypothetical protein NRI_0501 [Neorickettsia risticii str. Illinois] | 2 | IKVR |
|  |  |  |  |  |  |
| gi\|556054818\|gb\|JAB70454.1\| | 15,293 | **90** | putative ml domain-containing protein [Ixodes ricinus] | 2 | FEVDFVAER |
|  |  |  |  |  |  |
| gi\|442746893\|gb\|JAA65606.1\| | 17,198 | **99** | Putative nucleoside diphosphate kinase [Ixodes ricinus] | 2 | NIIHGSDSLPSAEK |
|  |  |  |  | 2 | FMQASEELLQK |
|  |  |  |  | 2 | EIALWFNEK |
|  |  |  |  |  |  |
| gi\|215510729\|gb\|EEC20182.1\| | 14,859 | **99** | fatty acid-binding protein FABP, putative [Ixodes scapularis] | 1 | TSTLLK |
|  |  |  |  | 2 | LAQTSKPSVELK |
|  |  |  |  | 2 | QFGDKEVTIVR |
|  |  |  |  | 2 | LNDVVAIR |
|  |  |  |  |  |  |
| gi\|215491972\|gb\|EEC01613.1\| | 54,929 | **99** | protein disulfide isomerase, putative [Ixodes scapularis] | 2 | KYGYK |
|  |  |  |  | 2 | HATDELK |
|  |  |  |  | 1 | EAGGIVK |
|  |  |  |  | 1 | SLVTESTK |
|  |  |  |  | 1 | LAPEYEK |
|  |  |  |  | 2 | EHDDFIK |
|  |  |  |  | 2 | SEPVPETNDGPVK |
|  |  |  |  | 1 | VAVAENFK |
|  |  |  |  |  |  |
| gi\|442747295\|gb\|JAA65807.1\| | 54,708 | **99** | Putative erp60 [Ixodes ricinus] | 2 | GGEFSADYNGPR |
|  |  |  |  | 2 | DASLHENFLK |
|  |  |  |  | 2 | TNDPPVPLIK |
|  |  |  |  | 2 | TLADEDILVVK |
|  |  |  |  |  |  |
| gi\|442758229\|gb\|JAA71273.1\| | 39,174 | **99** | Putative fructose-biphosphate aldolase [Ixodes ricinus] | 2 | ATVTTLQR |
|  |  |  |  | 1 | ALQASALK |
|  |  |  |  | 2 | ATAEAIVAPGK |
|  |  |  |  | 1 | ATAEAIVAPGK |
|  |  |  |  | 2 | LQGIGVENTEENRR |
|  |  |  |  | 2 | LQGIGVENTEENR |
|  |  |  |  | 2 | GILAADESTSTMGK |
|  |  |  |  |  |  |
| gi\|215504607\|gb\|EEC14101.1\| | 39,448 | **99** | fructose 1,6-bisphosphate aldolase, putative [Ixodes scapularis] | 2 | YVAGSIDSLAADR |
|  |  |  |  | 2 | VTEQVLAAVYK |
|  |  |  |  | 1 | VTEQVLAAVYK |
|  |  |  |  | 1 | QYR |
|  |  |  |  |  |  |
| gi\|215510720\|gb\|EEC20173.1\| | 79,111 | **90** | elongation factor, putative [Ixodes scapularis] | 2 | AYLPVNESFGFTADLR |

| Accession | Mass kDa | Score (%) | Description | z | Peptide |
| --- | --- | --- | --- | --- | --- |
| gi\|556054634\|gb\|JAB70362.1\| | 161,482 | 99 | putative vitellogenin-2 [Ixodes ricinus] | 2 | GAVHYLER |
|  |  |  |  | 2 | YASDTNQPEHR |
|  |  |  |  | 1 | ADDSYLPR |
|  |  |  |  | 3 | FAKPLDHTK |
|  |  |  |  | 2 | GEGEESPGDKLK |
|  |  |  |  | 1 | GSLLSK |
|  |  |  |  | 2 | ENAEGKPLGINR |
|  |  |  |  | 1 | SETAYLR |
|  |  |  |  | 1 | EVYLSAK |
|  |  |  |  | 2 | EVYLSAK |
|  |  |  |  | 2 | KFALDQTQDAK |
|  |  |  |  | 2 | DYVYHYNGK |
|  |  |  |  | 2 | GVLSIFQLDLVK |
|  |  |  |  | 2 | FSEGKLEEAELSK |
|  |  |  |  | 2 | LLNQVVGPQPGSTK |
|  |  |  |  | 2 | SQVILSSGYDPK |
|  |  |  |  | 2 | LNNLAVFHEGK |
|  |  |  |  | 2 | YVTTFDLSTDKDK |
|  |  |  |  | 2 | GLHDYWYESDDR |
|  |  |  |  | 2 | FYYATQNPEWHPR |
|  |  |  |  | 3 | EDKLTDDEAEHFLGK |
|  |  |  |  | 2 | LTDDEAEHFLGK |
|  |  |  |  | 2 | VQLFYDR |
|  |  |  |  | 1 | FESWGLDK |
|  |  |  |  | 3 | EVEDALPITDRDYDHVYGR |
|  |  |  |  | 2 | FPEPEWER |
|  |  |  |  | 1 | DIALPVYK |
|  |  |  |  | 2 | FTLDLER |
|  |  |  |  | 1 | TNPDLYLLK |
|  |  |  |  | 3 | TLDLEEVHDANTDTQLPDDLEK |
|  |  |  |  | 3 | FISGLNHLAALEYEDSDIKDVHSK |
|  |  |  |  | 2 | YVVPMWNDVSR |
|  |  |  |  | 2 | DYFHGYSFETVSLK |
|  |  |  |  | 3 | ELQFTLVPAAQDTTTEVEVDLGYK |
|  |  |  |  | 2 | SAALWALVR |
|  |  |  |  | 2 | NYFGEVLGYGLNVK |
|  |  |  |  | 2 | ELQFTLVPAAQDTTTEVEVDLGYK |
|  |  |  |  | 2 | LSLDLYGHAFDTWEFDESILQELSK |
|  |  |  |  |  |  |
| gi\|556054820\|gb\|JAB70455.1\| | 175,61 | 99 | putative vitellogenin-2 [Ixodes ricinus] | 2 | GAVHYLER |
|  |  |  |  | 2 | YASDTNQPEHR |
|  |  |  |  | 1 | ADDSYLPR |
|  |  |  |  | 3 | FAKPLDHTK |
|  |  |  |  | 2 | GEGEESPGDKLK |
|  |  |  |  | 1 | GSLLSK |
|  |  |  |  | 2 | ENAEGKPLGINR |
|  |  |  |  | 1 | AFIQTTK |
|  |  |  |  | 1 | SETAYLR |
|  |  |  |  | 1 | EVYLSAK |
|  |  |  |  | 2 | EVYLSAK |
|  |  |  |  | 2 | KFALDQTQDAK |
|  |  |  |  | 1 | LTTDVEYK |
|  |  |  |  | 2 | DYVYHYNGK |
|  |  |  |  | 2 | GVLSIFQLDLVK |
|  |  |  |  | 2 | FSEGKLEEAELSK |
|  |  |  |  | 2 | LLNQVVGPQPGSTK |
|  |  |  |  | 2 | SQVILSSGYDPK |
|  |  |  |  | 2 | LNNLAVFHEGK |
|  |  |  |  | 2 | YVTTFDLSTDKDK |
|  |  |  |  | 2 | GLHDYWYESDDR |
|  |  |  |  | 2 | FYYATQNPEWHPR |
|  |  |  |  | 3 | EDKLTDDEAEHFLGK |
|  |  |  |  | 2 | LTDDEAEHFLGK |
|  |  |  |  | 2 | VQLFYDR |
|  |  |  |  | 1 | FESWGLDK |
|  |  |  |  | 3 | EVEDALPITDRDYDHVYGR |
|  |  |  |  | 2 | FPEPEWER |
|  |  |  |  | 1 | DIALPVYK |
|  |  |  |  | 2 | FTLDLER |
|  |  |  |  | 1 | TNPDLYLLK |
|  |  |  | Accession  Mass  kDa  Score (%)  Description  z  Peptides  gi\|215494047\|gb\|EEC03688.1\|  71,165  99  heat shock protein, putative [Ixodes scapularis]  1  ITITNDK  1  LIGDAAK  1  NTTIPTR  1  STLEPVEK  2  SQVHDIVLVGGSTR  1  DNNLLGK  2  VEIIANDQGNR  2  STAGDTHLGGEDFDNR  2  WLDTNQLADK  1  EIAEAYLGK  2  TTPSYVAFTDTER  2  NALESYSFNIK  2  IINEPTAAAIAYGLDK  2  FEELNADLFR  2  QKELEQVCNPIITK  gi\|442760941\|gb\|JAA72629.1\|  34,898  **99**  Putative carbonic anhydrase, partial [Ixodes ricinus]  2  APTDGPILK  1  APTDGPILK  2  QSPIDIVQK  2  NVQSDPFLAENPLR  2  EPIEVSQEQFEAFR  gi\|442747467\|gb\|JAA65893.1\|  52,115  **99**  Putative erp60 [Ixodes ricinus]  2  TNDPPVPLIK  2  TLADEDVLVVK  2  MTNDFSVENLEK  2  FLEEYLAGNVK  gi\|442747295\|gb\|JAA65807.1\|  54,708  **99**  Putative erp60 [Ixodes ricinus]  2  TNDPPVPLIK  2  MTNDFSVENLEK  2  TLADEDILVVK  2  FLEEYLAGNVK  gi\|122555\|sp\|P02073.1\|HBB_ALCAA  16,223  **99**  RecName: Full=Hemoglobin subunit beta  1  MLTAEEK  2  VVTGVANALAHR  2  LHVDPENFR  3  LHVDPENFR  2  VLDAFSEGLK  1  VLDAFSEGLK  2  LLVVYPWTQR  gi\|122678\|sp\|P21380.1\|HBB_RANTA  16,167  **99**  RecName: Full=Hemoglobin subunit beta  2  VVTGVANALAHR  2  LHVDPENFR  3  LHVDPENFR  2  AAVTGFWGKVK  1  AAVTGFWGK  2  LLVVYPWTQR  gi\|556054634\|gb\|JAB70362.1\|  161,482  **99**  putative vitellogenin-2 [Ixodes ricinus]  1  GSLLSK  2  ENAEGKPLGINR  1  DIALPVYK  1  YSFTK  1  SETAYLR  1  EVYLSAK  2  KFALDQTQDAK  2  GVLSIFQLDLVK  2  FALDQTQDAK  2  LLNQVVGPQPGSTK  2  SQVILSSGYDPK  2  LNNLAVFHEGK  2  YVTTFDLSTDKDK  2  EVEDALPITDR  2  FYYATQNPEWHPR  2  GLHDYWYESDDR  3  VHHIAQSFPQSDEAESLDELK  2  LTDDEAEHFLGK  3  EVEDALPITDRDYDHVYGR  1  FESWGLDK  2  FPEPEWER  2  YDYGGSSVFAQVR  2  TNPDLYLLK  3  TLDLEEVHDANTDTQLPDDLEK  2  TLDLEEVHDANTDTQLPDDLEK  1  IAAFVNVLK  3  FISGLNHLAALEYEDSDIKDVHSK  3  DYFHGYSFETVSLK  2  NYFGEVLGYGLNVK  2  ELQFTLVPAAQDTTTEVEVDLGYK  gi\|556054820\|gb\|JAB70455.1\|  175,61  **99**  putative vitellogenin-2 [Ixodes ricinus]  1  GSLLSK  2  ENAEGKPLGINR  1  AFIQTTK  1  DIALPVYK  1  YSFTK  1  SETAYLR  1  EVYLSAK  2  KFALDQTQDAK  2  LHVVSQVPPVEK  2  FALDQTQDAK  2  LLNQVVGPQPGSTK  2  SQVILSSGYDPK  2  LNNLAVFHEGK  2  YVTTFDLSTDKDK  2  EVEDALPITDR  2  FYYATQNPEWHPR  2  GLHDYWYESDDR  3  VHHIAQSFPQSDEAESLDELK  2  LTDDEAEHFLGK  3  EVEDALPITDRDYDHVYGR  1  FESWGLDK  2  FPEPEWER  2  YDYGGSSVFAQVR  2  TNPDLYLLK  3  TLDLEEVHDANTDTQLPDDLEK  2  TLDLEEVHDANTDTQLPDDLEK  1  IAAFVNVLK  3  FISGLNHLAALEYEDSDIKDVHSK  3  DYFHGYSFETVSLK  2  NYFGEVLGYGLNVK  2  ELQFTLVPAAQDTTTEVEVDLGYK  gi\|215508461\|gb\|EEC17915.1\|  151,678  **95**  hemelipoglycoprotein precursor, putative [Ixodes scapularis]  3  KKSKNRHR  1  GSLLSK  2  LHVVSQVPPVEK  2  LNNLAVFHEGK  2  SQLLISSGYDPK  2  YDYGGATTFGQIR  gi\|215504084\|gb\|EEC13578.1\|  177,654  **92**  hemelipoglycoprotein precursor, putative [Ixodes scapularis]  2  HPELVR  1  GSLLSK  1  DIALPVYK  1  YSFTK  3  EVEDALPVTDREYDHVYGR  3  TLDLEEVHDANTDTQLPEDLER  gi\|215505979\|gb\|EEC15473.1\|  56,912  **99**  protein disulfide isomerase, putative [Ixodes scapularis]  3  VDATIETQLAETYEVR  1  TFVDASK  2  GTNEAVEYNGER  1  TLEGLSK  2  RPDLVIAK  2  FDGTANELEHTK  2  QLVDESSDIK  2  AAPEEVTEEEEEEEDKEDK  gi\|215495481\|gb\|EEC05122.1\|  60,484  **99**  chaperonin subunit, putative [Ixodes scapularis]  2  VGGSSEVEVNEK  2  VNDALNATR  1  TGVAIVK  1  DGVITVK  1  VGLQVAAVK  gi\|215495481\|gb\|EEC05122.1\|  60,484  **99**  chaperonin subunit, putative [Ixodes scapularis]  1  DDTLLLK  2  DDTLLLK  1  LASGVALLK  3  LVQDVANNTNEEAGDGTTTATVLAR  2  VAQIRDEIDLSNSEYEK  2  NVILEQSWGSPK  2  VVEGSDDFGYDALR  2  GYISPYFINTSK  2  AAVEEGIVPGGGTALLR  2  NTYVNMISAGIIDPTK  2  KISNVQTLIPALELANTQR  2  TLSDELEVIEGMK  2  VEFQDALLLFSEK  3  TALLDASGVASLLTTAEAVVVELPKEEK  gi\|442757975\|gb\|JAA71146.1\|  47,874  **99**  Putative protein disulfide-isomerase [Ixodes ricinus]  2  VDATVETQLAETYEVR  2  LHNLLFVSK  2  KSPGFEDILK  1  NFDEVVFDK  2  SLMEGAVTSESVQSFVK  1  TFVQDVLDGK  2  TFVQDVLDGK  2  QSLLSQDLPEDWDR  1  ILEFFGLK  gi\|254590119\|gb\|ACT69481.1\|  4,326  **23**  hypothetical protein NRI_0501 [Neorickettsia risticii str. Illinois]  2  IKVR  gi\|556054818\|gb\|JAB70454.1\|  15,293  **90**  putative ml domain-containing protein [Ixodes ricinus]  2  FEVDFVAER  gi\|442746893\|gb\|JAA65606.1\|  17,198  **99**  Putative nucleoside diphosphate kinase [Ixodes ricinus]  2  NIIHGSDSLPSAEK  2  FMQASEELLQK  2  EIALWFNEK  gi\|215510729\|gb\|EEC20182.1\|  14,859  **99**  fatty acid-binding protein FABP, putative [Ixodes scapularis]  1  TSTLLK  2  LAQTSKPSVELK  2  QFGDKEVTIVR  2  LNDVVAIR  gi\|215491972\|gb\|EEC01613.1\|  54,929  **99**  protein disulfide isomerase, putative [Ixodes scapularis]  2  KYGYK  2  HATDELK  1  EAGGIVK  1  SLVTESTK  1  LAPEYEK  2  EHDDFIK  2  SEPVPETNDGPVK  1  VAVAENFK  gi\|442747295\|gb\|JAA65807.1\|  54,708  **99**  Putative erp60 [Ixodes ricinus]  2  GGEFSADYNGPR  2  DASLHENFLK  2  TNDPPVPLIK  2  TLADEDILVVK  gi\|442758229\|gb\|JAA71273.1\|  39,174  **99**  Putative fructose-biphosphate aldolase [Ixodes ricinus]  2  ATVTTLQR  1  ALQASALK  2  ATAEAIVAPGK  1  ATAEAIVAPGK  2  LQGIGVENTEENRR  2  LQGIGVENTEENR  2  GILAADESTSTMGK  gi\|215504607\|gb\|EEC14101.1\|  39,448  **99**  fructose 1,6-bisphosphate aldolase, putative [Ixodes scapularis]  2  YVAGSIDSLAADR  2  VTEQVLAAVYK  1  VTEQVLAAVYK  1  QYR  gi\|215510720\|gb\|EEC20173.1\|  79,111  **90**  elongation factor, putative [Ixodes scapularis]  2  AYLPVNESFGFTADLR | 3 | TLDLEEVHDANTDTQLPDDLEK |
|  |  |  |  | 3 | FISGLNHLAALEYEDSDIKDVHSK |
|  |  |  |  | 2 | YVVPMWNDVSR |
|  |  |  |  | 2 | DYFHGYSFETVSLK |
|  |  |  |  | 3 | ELQFTLVPAAQDTTTEVEVDLGYK |
|  |  |  |  | 2 | SAALWALVR |
|  |  |  |  | 2 | NYFGEVLGYGLNVK |
|  |  |  |  | 2 | ELQFTLVPAAQDTTTEVEVDLGYK |
|  |  |  |  | 2 | LSLDLYGHAFDTWEFDESILQELSK |
|  |  |  |  |  |  |
| gi\|215504084\|gb\|EEC13578.1\| | 177,654 | 99 | hemelipoglycoprotein precursor, putative [Ixodes scapularis] | 2 | GAVHYLER |
|  |  |  |  | 1 | ADDSYLPR |
|  |  |  |  | 1 | GSLLSK |
|  |  |  |  | 2 | LLNHVVGPQPGSSK |
|  |  |  |  | 2 | DYVYHYNGK |
|  |  |  |  | 3 | EVEDALPVTDREYDHVYGR |
|  |  |  |  | 1 | DIALPVYK |
|  |  |  |  | 3 | TLDLEEVHDANTDTQLPEDLER |
|  |  |  |  |  |  |
| gi\|442752113\|gb\|JAA68216.1\| | 55,243 | 99 | Putative imp dehydrogenase/gmp reductase [Ixodes ricinus] | 2 | DIDFIPIEDHNR |
|  |  |  |  | 2 | LPLVNEGGELVSLIAR |
|  |  |  |  | 1 | SYPLASK |
|  |  |  |  | 2 | VAQGVSGTIVDK |
|  |  |  |  | 1 | SLDVLK |
|  |  |  |  | 2 | HGFAGVPITENGK |
|  |  |  |  | 2 | GMGSLDAMQSTEGSGSLNR |
|  |  |  |  | 2 | SVSAQAEGGVHGLYSFEK |
|  |  |  |  | 2 | NLIEAGVDGLR |
|  |  |  |  | 2 | YPGLQIIGGNVVTTAQAK |
|  |  |  |  | 2 | DENKQLIVGDRQSATR |
|  |  |  |  | 2 | YVPYLITGIK |
|  |  |  |  |  |  |
| gi\|215491939\|gb\|EEC01580.1\| | 47,043 | 99 | IMP dehydrogenase, putative [Ixodes scapularis] | 2 | DIDFIPIEDHNR |
|  |  |  |  | 2 | LPLVNEGGELVSLIAR |
|  |  |  |  | 1 | DLTVASSK |
|  |  |  |  | 1 | SYPLASK |
|  |  |  |  | 2 | VTLSEANSLLQK |
|  |  |  |  | 2 | VAQGVSGTIVDK |
|  |  |  |  | 2 | HGFAGVPITENGK |
|  |  |  |  | 2 | SVSAQAEGGVHGLYSFEK |
|  |  |  |  | 2 | NLIEAGVDGLR |
|  |  |  |  | 2 | YVPYLITGIK |
|  |  |  |  |  |  |
| gi\|556066176\|gb\|JAB76114.1\| | 42,73 | 99 | putative imp dehydrogenase/gmp reductase, partial [Ixodes ricinus] | 2 | DIDFIPIEDHNR |
|  |  |  |  | 2 | LPLVNEGGELVSLIAR |
|  |  |  |  | 1 | DLTVASSK |
|  |  |  |  | 1 | SYPLASK |
|  |  |  |  | 2 | VTLSEANSLLQK |
|  |  |  |  | 2 | HGFAGVPITENGK |
|  |  |  |  | 2 | GMGSLDAMQSTEGSGSLNR |
|  |  |  |  | 2 | NLIEAGVDGLR |
|  |  |  |  | 2 | YPGLQIIGGNVVTTAQAK |
|  |  |  |  |  |  |
| gi\|442757299\|gb\|JAA70808.1\| | 57,181 | 99 | Putative catalase pediculus us corporis catalase [Ixodes ricinus] | 2 | TNVQTVGPR |
|  |  |  |  | 2 | LTSNIANHLK |
|  |  |  |  | 3 | FSTVGGESGSADTVRDPR |
|  |  |  |  | 2 | FSTVGGESGSADTVR |
|  |  |  |  | 2 | AGDLSGADPDYSIR |
|  |  |  |  | 2 | DLYNSIANK |
|  |  |  |  | 2 | GAGAFGYLEITHDITK |
|  |  |  |  | 2 | DPFLFPSFIHTQK |
|  |  |  |  | 2 | FYTEEGNWDLVGNNTPIFFIR |
|  |  |  |  |  |  |
| gi\|215491559\|gb\|EEC01200.1\| | 49,612 | 90 | D-3-phosphoglycerate dehydrogenase, putative [Ixodes scapularis] | 2 | NTQLIAHPK |
|  |  |  |  | 2 | WDRKTFMGNELYGK |
|  |  |  |  |  |  |
| gi\|215507080\|gb\|EEC16574.1\| | 59,844 | 90 | F0F1-type ATP synthase, alpha subunit, putative [Ixodes scapularis] | 2 | ILGQTTTSNLEETGR |
|  |  |  |  |  |  |
| gi\|215494293\|gb\|EEC03934.1\| | 37,593 | 99 | actin, putative [Ixodes scapularis] | 2 | EITALAPSTMK |
|  |  |  |  | 3 | IWHHTFYNELR |
|  |  |  |  | 2 | IWHHTFYNELR |
|  |  |  |  | 3 | VAPEEHPVLLTEAPLNPK |
|  |  |  |  | 1 | DLTDYLMK |
|  |  |  |  | 2 | SYELPDGQVITIGNER |
|  |  |  |  | 3 | TTGIVLDSGDGVSHTVPIYEGYALPHAILR |
|  |  |  |  |  |  |
| gi\|442760565\|gb\|JAA72441.1\| | 22,911 | 99 | Putative thioredoxin peroxidase, partial [Ixodes ricinus] | 1 | SVDETLR |
|  |  |  |  | 1 | DYFSK |
|  |  |  |  | 2 | LVQAFQYTDK |
|  |  |  |  | 1 | GLFIIDDK |
|  |  |  |  |  |  |
| gi\|556084942\|gb\|JAB82892.1\| | 20,241 | 99 | putative alpha crystallins [Ixodes ricinus] | 2 | GHGSQAVVESDPNK |
|  |  |  |  | 2 | SDETGSYVK |
|  |  |  |  | 1 | SDETGSYVK |
|  |  |  |  | 2 | HFAPEEITVK |
|  |  |  |  |  |  |
| gi\|122678\|sp\|P21380.1\|HBB_RANTA | 16,167 | 73 | RecName: Full=Hemoglobin subunit beta | 2 | LLVVYPWTQR |
|  |  |  |  |  |  |
| gi\|215510489\|gb\|EEC19942.1\| | 17,424 | 68 | small heat shock protein, putative [Ixodes scapularis] | 2 | YVLPEDVDPHTVTSSLSAGGLLAVEAPR |
|  |  |  |  |  |  |
| gi\|215495480\|gb\|EEC05121.1\| | 10,868 | 95 | heat shock protein [Ixodes scapularis] | 1 | DSDVLGK |
|  |  |  |  | 2 | VQSATVIAVGTGAR |
|  |  |  |  |  |  |
| gi\|442752151\|gb\|JAA68235.1\| | 14,78 | 90 | Putative pterin carbinolamine dehydratase pcbd [Ixodes ricinus] | 2 | LANFIETASK |
|  |  |  |  |  |  |
| gi\|215510489\|gb\|EEC19942.1\| | 17,424 | 74 | small heat shock protein, putative [Ixodes scapularis] | 2 | HFAPEEITVK |
|  |  |  |  |  |  |
| gi\|215510489\|gb\|EEC19942.1\| | 17,424 | 63 | small heat shock protein, putative [Ixodes scapularis] | 2 | HFAPEEITVK |
|  |  |  |  |  |  |
| gi\|39725997\|gb\|AAR29950.1\| | 47,377 | 98 | calreticulin [Ixodes minor] | 2 | QYGEETWGATK |
|  |  |  |  | 2 | IDNEVVESGELEK |
|  |  |  |  |  |  |
| gi\|215505979\|gb\|EEC15473.1\| | 56,912 | 99 | protein disulfide isomerase, putative [Ixodes scapularis] | 2 | TGPAAEDLK |
|  |  |  |  | 1 | TFVDASK |
|  |  |  |  | 2 | GTNEAVEYNGER |
|  |  |  |  | 1 | TLEGLSK |
|  |  |  |  | 2 | YKPEKDDLTPENVR |
|  |  |  |  | 2 | FDGTANELEHTK |
|  |  |  |  | 2 | QLVDESSDIK |
|  |  |  |  | 2 | AAPEEVTEEEEEEEDKEDK |
|  |  |  |  | 2 | FIDTDGEYGK |
|  |  |  |  |  |  |
| gi\|604824672\|gb\|JAC34673.1\| | 61,067 | 99 | putative chaperonin subunit [Amblyomma triste] | 1 | DGVTVAK |
|  |  |  |  | 2 | VGGSSEVEVNEK |
|  |  |  |  | 2 | VNDALNATR |
|  |  |  |  | 1 | DGVITVK |
|  |  |  |  | 2 | TLIDELEVIEGMK |
|  |  |  |  | 2 | GANPIEIR |
|  |  |  |  | 2 | VGLQVAAVK |
|  |  |  |  |  |  |
| gi\|556053694\|gb\|JAB69892.1\| | 37,871 | 90 | putative calreticulin [Ixodes ricinus] | 2 | GVGSRRRSTTR |
|  |  |  |  | 2 | EEFADGDGWTSR |
|  |  |  |  |  |  |
| gi\|442757321\|gb\|JAA70819.1\| | 56,896 | 99 | Putative protein disulfide-isomerase [Ixodes ricinus] | 2 | NFDEVVFDKEK |
|  |  |  |  | 2 | KSPGFEDILK |
|  |  |  |  | 1 | DGVVLFK |
|  |  |  |  | 2 | SPGFEDILK |
|  |  |  |  | 2 | QLAPIYDELAEK |
|  |  |  |  | 2 | SLMEGAVTSESVQSFVK |
|  |  |  |  | 2 | TFVQDVLDGK |
|  |  |  |  | 2 | QSLLSQDLPEDWDR |
|  |  |  |  |  |  |
| gi\|215495481\|gb\|EEC05122.1\| | 60,484 | 99 | chaperonin subunit, putative [Ixodes scapularis] | 2 | LASGVALLK |
|  |  |  |  | 1 | DDTLLLK |
|  |  |  |  | 3 | VAQIRDEIDLSNSEYEKEK |
|  |  |  |  | 3 | LVQDVANNTNEEAGDGTTTATVLAR |
|  |  |  |  | 2 | NVILEQSWGSPK |
|  |  |  |  | 2 | VVEGSDDFGYDALR |
|  |  |  |  | 2 | AAVEEGIVPGGGTALLR |
|  |  |  |  | 2 | GYISPYFINTSK |
|  |  |  |  | 2 | VEFQDALLLFSEK |
|  |  |  |  |  |  |
| gi\|556063403\|gb\|JAB74737.1\| | 44,872 | 92 | putative glutamine synthetase [Ixodes ricinus] | 1 | AIEAAIER |
|  |  |  |  | 2 | VTEVLVR |
|  |  |  |  | 1 | VTEVLVR |
|  |  |  |  | 3 | LTDLAGKPTLFQYLDLPQPSDK |
|  |  |  |  |  |  |
| gi\|215498464\|gb\|EEC07958.1\| | 16,858 | 90 | actin depolymerizing factor, putative [Ixodes scapularis] | 2 | LILMTWCPESSKIK |
|  |  |  |  | 2 | NATYSEFLEELQK |
|  |  |  |  |  |  |
| gi\|556054818\|gb\|JAB70454.1\| | 15,293 | 90 | putative ml domain-containing protein [Ixodes ricinus] | 2 | FEVDFVAER |
|  |  |  |  |  |  |
| gi\|442746893\|gb\|JAA65606.1\| | 17,198 | 99 | Putative nucleoside diphosphate kinase [Ixodes ricinus] | 2 | NIIHGSDSLPSAEK |
|  |  |  |  | 2 | FMQASEELLQK |
|  |  |  |  | 2 | EIALWFNEK |
|  |  |  |  |  |  |
| gi\|122555\|sp\|P02073.1\|HBB_ALCAA | 16,223 | 99 | RecName: Full=Hemoglobin subunit beta | 1 | HLDDLK |
|  |  |  |  | 2 | MLTAEEK |
|  |  |  |  | 3 | VVTGVANALAHR |
|  |  |  |  | 2 | VVTGVANALAHR |
|  |  |  |  | 2 | LHVDPENFR |
|  |  |  |  | 2 | RVLDAFSEGLK |
|  |  |  |  | 2 | VLDAFSEGLK |
|  |  |  |  | 1 | VLDAFSEGLK |
|  |  |  |  | 2 | LLVVYPWTQR |
|  |  |  |  |  |  |
| gi\|376324229\|gb\|AFB21470.1\| | 99,128 | 22 | DNA polymerase I [Rickettsia canadensis str. CA410] | 2 | ASAKTNSYSTGVEILK |
|  |  |  |  |  |  |
| gi\|442749105\|gb\|JAA66712.1\| | 33,32 | 43 | Putative c1-tetrahydrofolate synthase [Ixodes ricinus] | 2 | EDSNVYIR |
