# Supplementary material for: Ixodes ricinus and Its Endosymbiont Midichloria mitochondrii: A Comparative Proteomic Analysis of Salivary Glands and Ovaries
Source: PLoS One. 2015 Sep 23;10(9):e0138842. doi: 10.1371/journal.pone.0138842 (PMC4580635; doi:10.1371/journal.pone.0138842)
Supplement: S3 Table — (DOCX) [file pone.0138842.s003.docx]

| **Spot** | **Accession** | **Mass** | **Score (%)** | **Description** | **z** | **Peptides** |
| --- | --- | --- | --- | --- | --- | --- |
| 1 | gi\|442756551\|gb\|JAA70434.1\| | 72,595 | 99 | Putative heat shock 70 kDa protein 5 [Ixodes ricinus] | 2 | ITINNDQNR |
|  |  |  |  |  | 1 | TIEEAVDEK |
|  |  |  |  |  | 3 | WLEQHSDADAEELKEQK |
|  |  |  |  |  | 2 | QLADTVQPIVAK |
|  |  |  |  |  | 2 | NELESYAYSLK |
|  |  |  |  |  |  |  |
| 2 | / | / | / | Not detectable | / | / |
|  |  |  |  |  |  |  |
| 3 | gi\|322422107\|gb\|ADX01224.1\| | 16,038 | 90 | Beta actin [Ixodes ricinus] | 2 | DSYVGDEAQSK |
|  |  |  |  |  |  |  |
| 4 | gi\|215497327\|gb\|EEC06821.1\| | 21,493 | 90 | Enolase, putative [Ixodes scapularis] | 2 | HIADLAGNSK |
|  |  |  |  |  |  |  |
| 5 | gi\|442753241\|gb\|JAA68780.1\| | 47,145 | 99 | Putative enolase [Ixodes ricinus] | 2 | VNQIGTVTESIR |
|  |  |  |  |  | 2 | NPNSNPGDFLER |
|  |  |  |  |  | 2 | AAVPSGASTGIHEALELR |
|  |  |  |  |  | 2 | IDIGMDVAASEFYK |
|  |  |  |  |  | 2 | MPITKIFARQIFDSR |
|  |  |  |  |  | 2 | EALELIMSAISAAGYTGK |
|  |  |  |  |  |  |  |
| 6 | gi\|215491972\|gb\|EEC01613.1\| | 54,929 | 98 | Protein disulfide isomerase, putative [Ixodes scapularis] | 1 | SLVTESTK |
|  |  |  |  |  | 2 | EHDDFIK |
|  |  |  |  |  | 1 | LNFAVSNK |
|  |  |  |  |  | 2 | GGEFSADYNGPR |
|  |  |  |  |  |  |  |
| 7 | gi\|442748259\|gb\|JAA66289.1\| | 10,741 | 20 | Putative 3-hydroxy-3-methylglutaryl-coa reductase [Borrelia spp] | 2 | EVRLFHSVLR |
|  |  |  |  |  | 2 | MRPTGTVLQTQR |
|  |  |  |  |  |  |  |
| 8 | gi\|597718071\|gb\|AHN19768.1\| | 66,150 | 75 | Serum albumin, partial [Cervus nippon] | 2 | VGEYGFQNALIVR |
|  |  |  |  |  | 1 | IVTDLTK |
|  |  |  |  |  | 1 | ADFAEVTK |
|  |  |  |  |  | 2 | TVMENFVAFVDK |
|  |  |  |  |  |  |  |
| 9 | / | / | / | Not detectable | / | / |
|  |  |  |  |  |  |  |
| 10 | gi\|442754645\|gb\|JAA69482.1\| | 36,782 | 82 | Putative heat shock protein [Ixodes ricinus] | 2 | LGDYPNAR |
|  |  |  |  |  | 2 | IPDTLAK |
