# Supplementary material for: Ixodes ricinus and Its Endosymbiont Midichloria mitochondrii: A Comparative Proteomic Analysis of Salivary Glands and Ovaries
Source: PLoS One. 2015 Sep 23;10(9):e0138842. doi: 10.1371/journal.pone.0138842 (PMC4580635; doi:10.1371/journal.pone.0138842)
Supplement: S4 Table — (DOCX) [file pone.0138842.s004.docx]

| Accession | Mass | Score (%) | Description | z | Peptides |
| --- | --- | --- | --- | --- | --- |
| gi\|442747467\|gb\|JAA65893.1\| | 52,115 | 98 | Putative erp60 [Ixodes ricinus] | 2 | GGEFSADYNGPR |
|  |  |  |  | 2 | DASLHENFLK |
|  |  |  |  | 2 | FLEEYLAGNVK |
|  |  |  |  |  |  |
| gi\|556065071\|gb\|JAB75571.1\| | 36,27 | 89 | putative actin-2 [Ixodes ricinus] | 3 | VAPEEHPVLLTEAPLNPK |
|  |  |  |  | 2 | SYELPDGQVITIGNER |
|  |  |  |  |  |  |
| gi\|6841058\|gb\|AAF28881.1\| | 29,518 | 25 | unknown [Borrelia hermsii]; | 2 | NTPVRFYLNDK |
