# Supplementary material for: Ixodes ricinus and Its Endosymbiont Midichloria mitochondrii: A Comparative Proteomic Analysis of Salivary Glands and Ovaries
Source: PLoS One. 2015 Sep 23;10(9):e0138842. doi: 10.1371/journal.pone.0138842 (PMC4580635; doi:10.1371/journal.pone.0138842)
Supplement: S5 Table — (DOCX) [file pone.0138842.s005.docx]

| Accession | Mass | Score (%) | Description | z | Peptides |
| --- | --- | --- | --- | --- | --- |
| / | 100,58 | 84 | Flagellar protein FLID OS=Midichloria mitochondrii | 3 | IEVNIKRELANIAGIR |
|  |  |  |  | 3 | IDVEEIKKIEVNIK |
|  |  |  |  | 2 | SAHIKSLFNR |
|  |  |  |  | 3 | AKLTEPVTR |
|  |  |  |  | 2 | IDVEEIKKIEVNIK |
|  |  |  |  | 3 | KISSAPLDNIPEEVK |
|  |  |  |  | 2 | KISSAPLDNIPEEVK |
|  |  |  |  | 2 | ANFKDIGIDFKQEK |
|  |  |  |  | 3 | IKFDGVAKSFK |
|  |  |  |  | 3 | DEVNNRHKLALISK |
|  |  |  |  | 1 | INSAKAGVR |
|  |  |  |  | 2 | AFGVDTIIGK |
|  |  |  |  | 1 | SDAFRR |
|  |  |  |  |  |  |
| gi\|442747467\|gb\|JAA65893.1\| | 52,115 | 58 | Putative erp60 [Ixodes ricinus] | 2 | GGEFSADYNGPR |
|  |  |  |  | 2 | DASLHENFLK |
|  |  |  |  | 2 | FLEEYLAGNVK |
